# Supplementary material for: Cation vacancy stabilization of single-atomic-site Pt1/Ni(OH)x catalyst for diboration of alkynes and alkenes
Source: Nat Commun. 2018 Mar 8;9:1002. doi: 10.1038/s41467-018-03380-z (PMC5843605; doi:10.1038/s41467-018-03380-z)
Supplement: Supplementary file 2 — Supplementary Information [file 41467_2018_3380_MOESM2_ESM.pdf]

Supplementary Information

Cation Vacancy Stabilization of Single-Atomic-Site $Pt_1/Ni(OH)_x$ Catalyst for Diboration of Alkynes and Alkenes

Zhang et al.

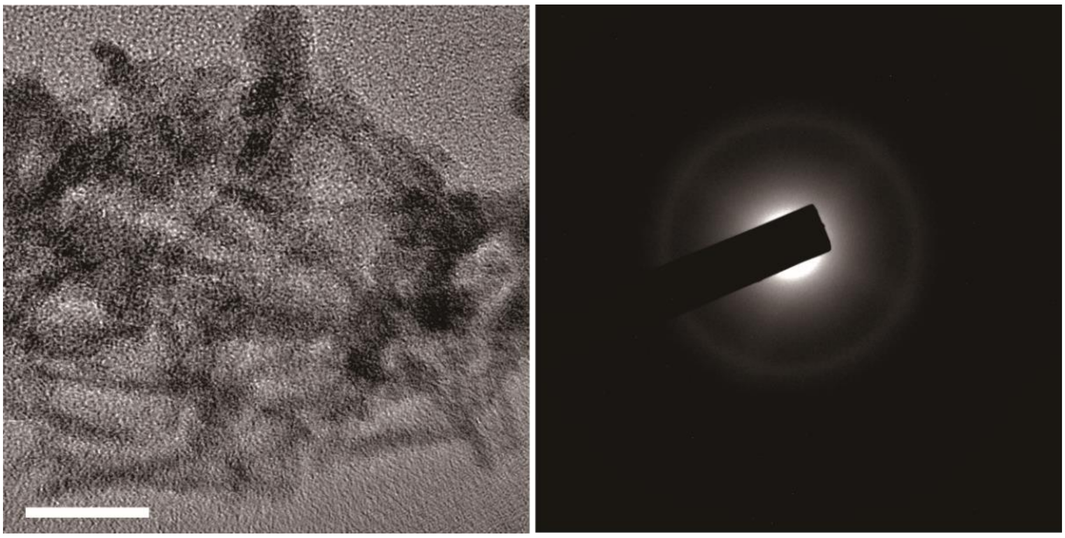

Supplementary Figure 1 | The HR-TEM image and corresponding SEAD pattern of $Ni(OH)_x$ nanoboards. The diffraction disks observed in the SEAD pattern manifest the polycrystalline structure of the $Ni(OH)_x$ nanoboards. Scale bar, 20 nm.

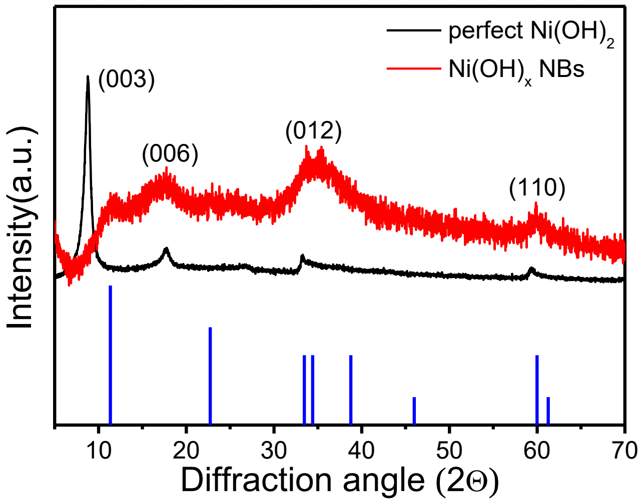

Supplementary Figure 2 | XRD patterns of $Ni(OH)_x$ NBs in comparison to the perfect $Ni(OH)_2$. It can be seen that all the diffraction peaks of $Ni(OH)_x$ NBs are similar to that of the perfect $Ni(OH)_2$, which are consistent with a hexagonal layered structure α-$Ni(OH)_2$•$0.75H_2O$ with lattice parameters of $a = b = 3.08$ Å and $c = 23.41$ Å (JCPDS 38-0715)[1]. The strong diffraction peak from (012) planes clearly reveals that $Ni(OH)_x$ NBs possess a preferred (012) orientation.

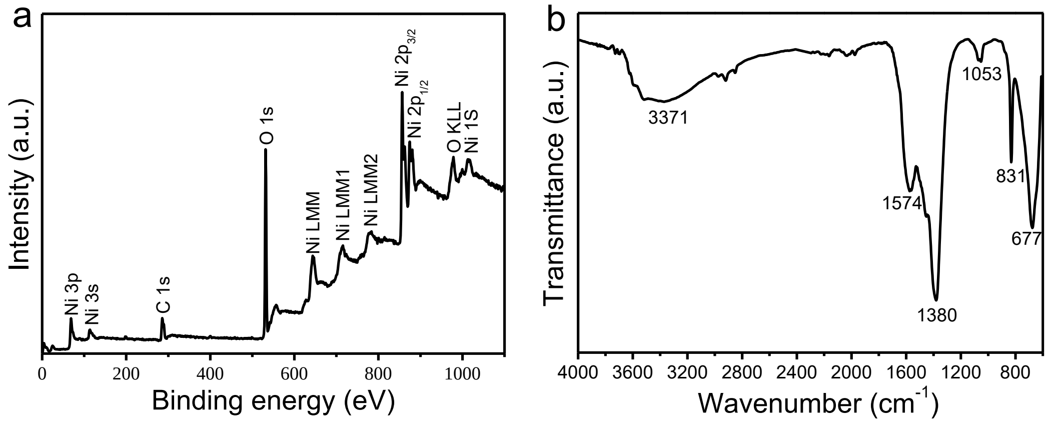

Supplementary Figure 3 | The XPS and FT-IR spectra of $Ni(OH)_x$ nanoboards. (**a**) In this typical XPS spectrum, two main peaks at 855.7 and 873.4 eV correspond to Ni $2p_{3/2}$ and Ni $2p_{1/2}$, respectively, yielding a spin-energy separation of 17.7 eV characteristic of the nickel hydroxide phase. In addition, the O 1s spectrum with a strong peak at 531.0 eV is associated with bound hydroxide groups (OH^-). (**b**) As can be seen from this FT-IR spectrum, a broad band at around 3,371 cm^{-1} can be assigned to the O–H vibration of hydrogen-bonded hydroxyl groups from the metal hydroxide and intercalated water molecule. The band at 1574 cm^{-1} corresponds to the bending mode of the interlayer water molecule. Two bands at 1380 and 831 cm^{-1} are characteristic of nitrate ions. The weak band at 1053 cm^{-1} indicates the presence of a few carbonate ions. Finally, the band at 677 cm^{-1} can be ascribed to the bending mode of hydroxyl groups from the metal hydroxide. Above XPS and FT-IR data together confirm that the as-synthesized sample is a typical nickel hydroxide[2,3].

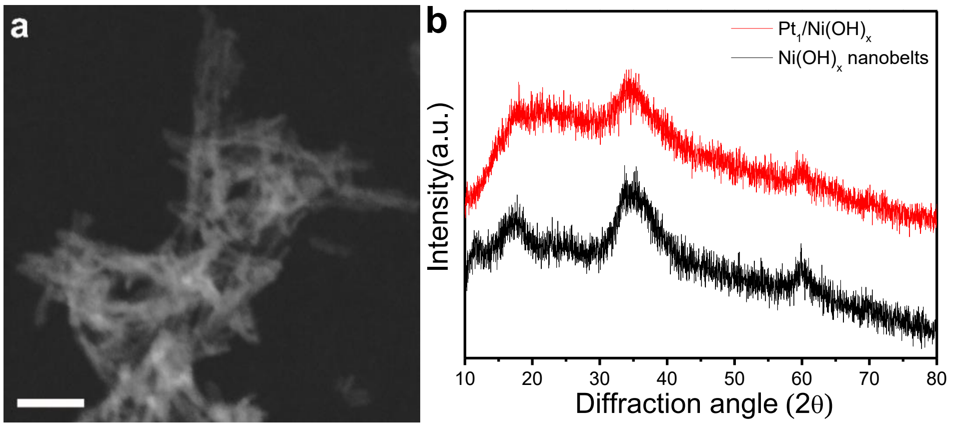

Supplementary Figure 4 | STEM image and XRD pattern of $Pt_1/Ni(OH)_x$. (**a**) The STEM image suggests no Pt nanoparticles form on the $Ni(OH)_x$ nanoboards. Scale bar, 20 nm. (**b**) As can be seen, the XRD pattern of $Pt_1/Ni(OH)_x$ is similar to $Ni(OH)_x$ nanoboards without peaks attributed to Pt nanoparticles detected.

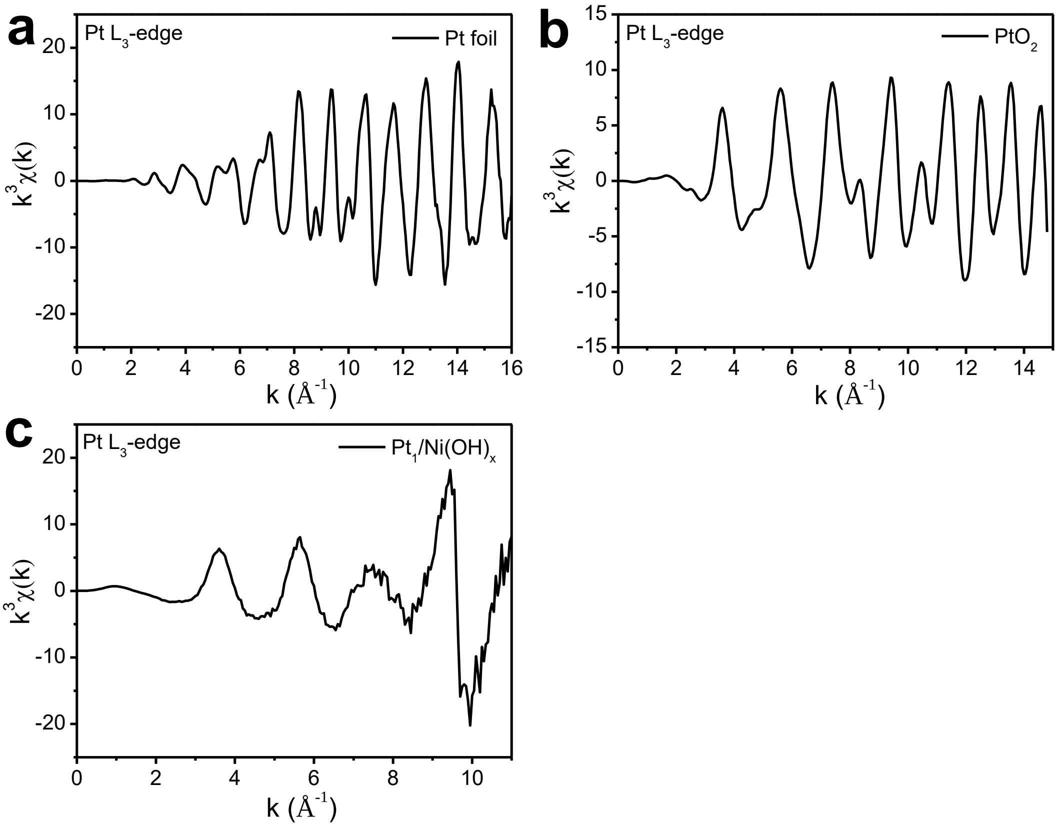

Supplementary Figure 5 | Corresponding EXAFS in k-space at the Pt-L_3 edge. The k^3-weighted EXAFS in k-space for the Pt foil (**a**), PtO_2 (**b**) and $Pt_1/Ni(OH)_x$ (**c**) at the Pt-L_3 edge.

Supplementary Table 1 | Control experiments for diboration reactions.

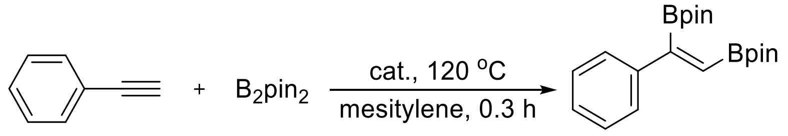

1a **2a** **3aa**

Entry	Cat.	Conv. (%)^a	Sel. (%)^b	TOF (h^{-1})
1	$Pt_1/Ni(OH)_x$	97	99	3233
2	$Ni(OH)_x$ NBs	0	0	0
3	$Pt/Ni(OH)_2$	22	99	680
4	perfect $Ni(OH)_2$	0	0	0

Standard reaction conditions: substrate **1a** (0.50 mmol) and **2a** (0.50 mmol), catalyst: $Ni(OH)_x$ NB (6.5 mg), $Pt_1/Ni(OH)_x$ (6.5 mg, Pt/substrate = 0.1%), $Ni(OH)_2$ (10.8 mg), $Pt/Ni(OH)_2$ (10.8 mg, Pt/substrate = 0.1%), mesitylene (2.0 mL) as solvent, T = 120 °C, t = 0.3 h. ^{a} Determined by gas chromatography (GC) analysis with dodecane as internal standard. ^{b} Determined by GC-MS analysis.

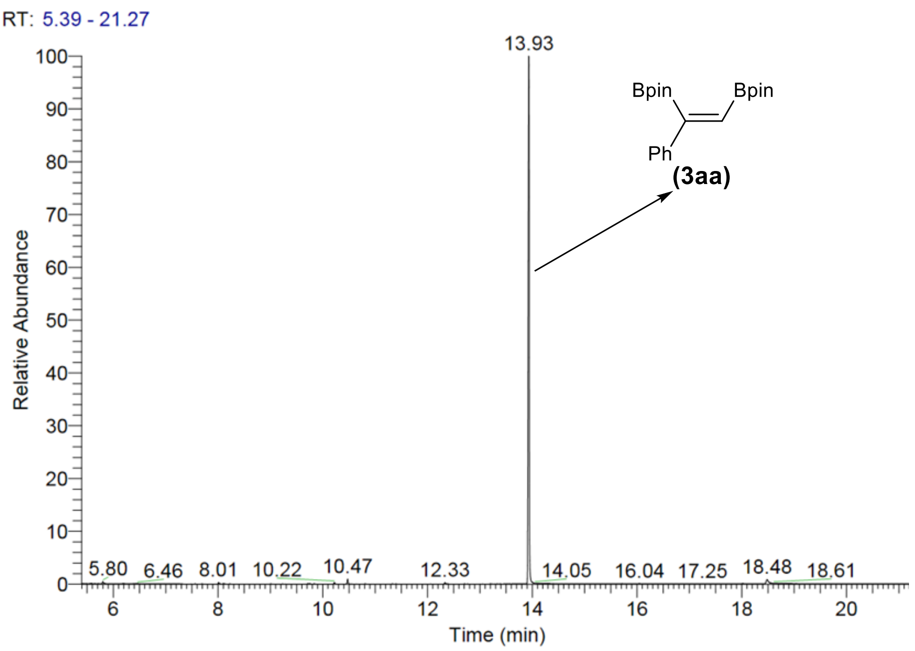

Supplementary Figure 6 | GC-MS analysis of reaction mixture from $Pt_1/Ni(OH)_x$ catalyzed diboration of phenylacetylene. It is obvious that there is a main peak of target macular (**3a**) at 13.93 min and few other apparent peaks of by-products in this GC-MS spectrum, indicating the excellent selectivity of the $Pt_1/Ni(OH)_x$ catalyst.

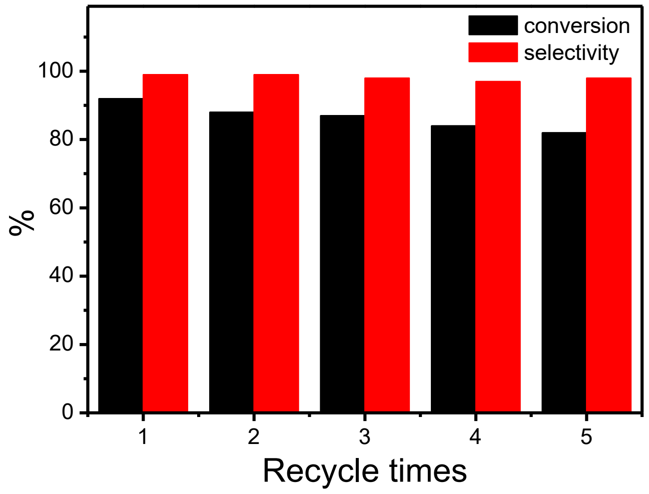

Supplementary Figure 7 | Recycling test of the $Pt_1/Ni(OH)_x$ catalyst. The catalyst was recovered by centrifugation of the reaction mixture and used for next recycling run directly. Reaction conditions: phenylacetylene (0.5 mmol, 54.9 μL), B_2pin_2 (0.5 mmol, 126.7 mg) and $Pd_1/Ni(OH)_x$ (Pt/substrate = 0.1 %) in mesitylene (2.0 mL) at 120 °C for 20 min.

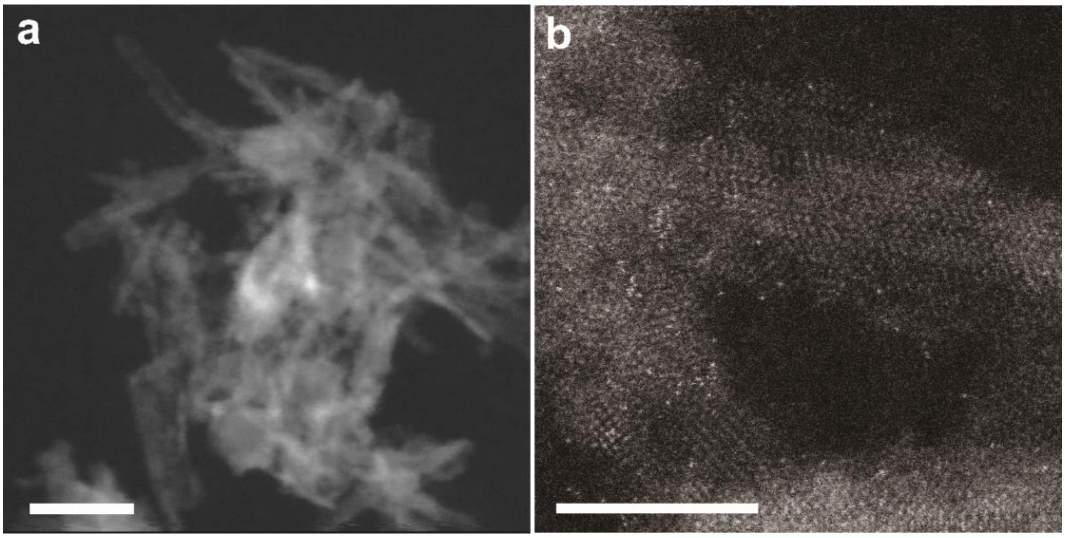

Supplementary Figure 8 | STEM and AC HAADF-STEM images of the $Pt_1/Ni(OH)_x$ catalyst after five recycle experiments. (**a**) STEM image shows that the recovered $Pt_1/Ni(OH)_x$ catalyst remains nanoboard morphology without the formation of Pt nanoparticles. Scale bar, 20 nm. (**b**) AC HAADF-STEM image suggests that Pt components still exist at single-atomic sites. Scale bar, 5 nm.

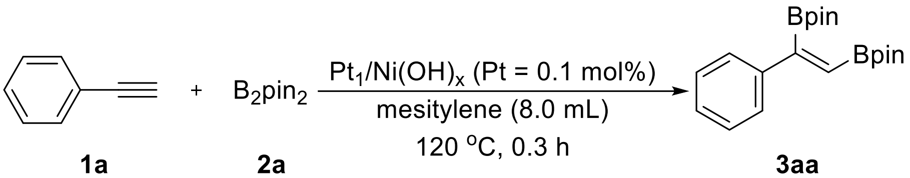

1a 2.0 mmol

2a 2.0 mmol

3aa isolated yield = 93%

Supplementary Figure 9 | Expansion of the scale of the diboration reaction over $Pt_1/Ni(OH)_x$. Standard reaction conditions: substrate **1a** (2.0 mmol) and **2a** (2.0 mmol), catalyst: $Pt_1/Ni(OH)_x$ (26.5 mg, Pt/substrate = 0.1%), mesitylene (8.0 mL) as solvent, T = 120 °C, t = 0.3 h. The product **3aa** was isolated in 93 % yield by flash column chromatography on silica gel with *n*-hexane/ethyl acetate as eluent.

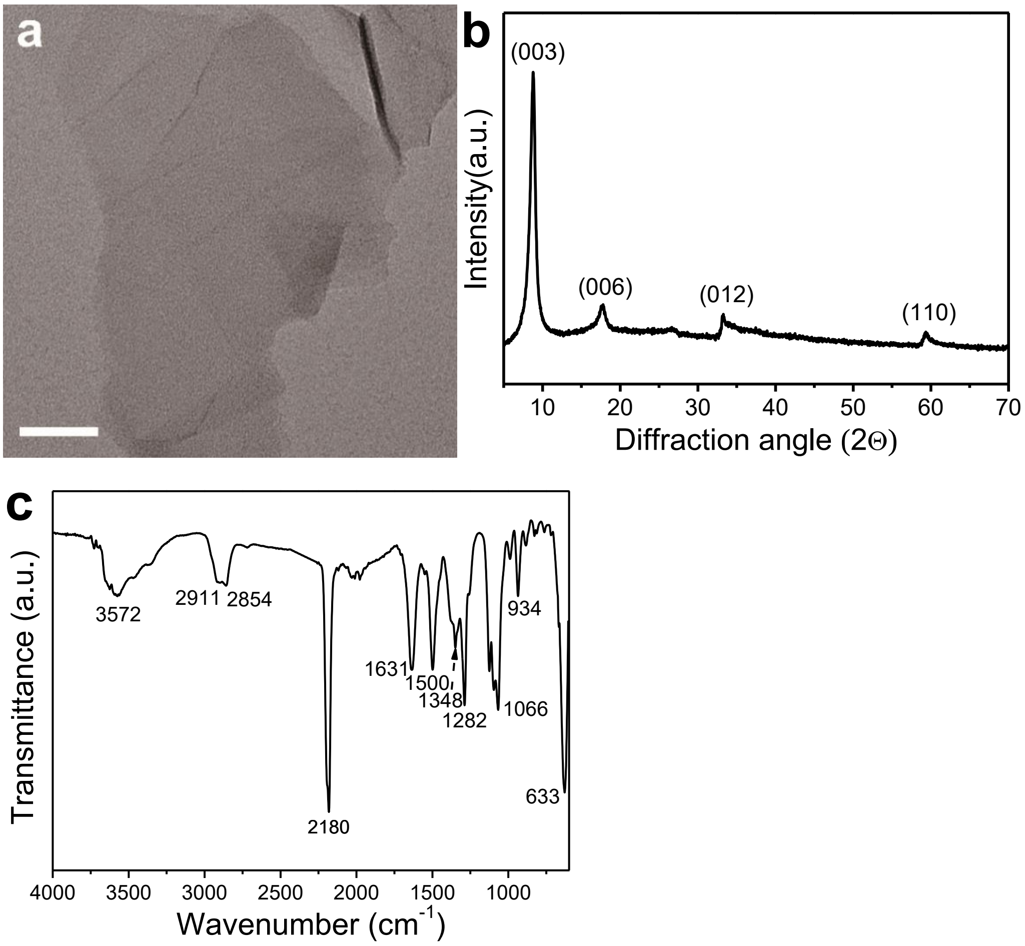

Supplementary Figure 10 | Characterization of the as-synthesized perfect $Ni(OH)_2$ material. (**a**) TEM image of the perfect $Ni(OH)_2$. Scale bar, 50 nm. (**b**) XRD pattern of the perfect $Ni(OH)_2$. The strongest reflection at 2θ of 8.8° in the XRD pattern suggests that this perfect $Ni(OH)_2$ is characteristic of α-$Ni(OH)_2$ (JCPDS 38-0715) with good crystallinity[1]. (**c**) The FT-IR spectrum of the perfect $Ni(OH)_2$.

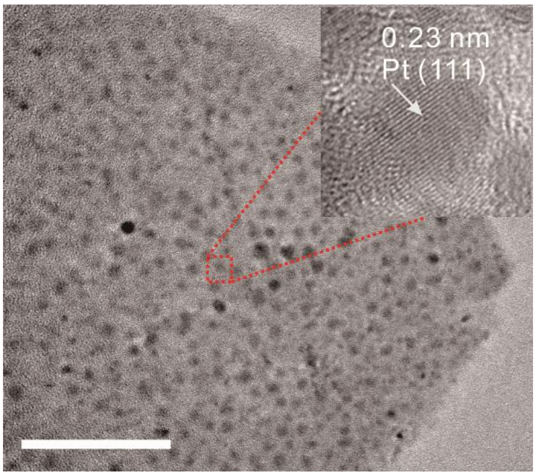

Supplementary Figure 11 | Representative HR-TEM image of the obtained Pt/$Ni(OH)_2$ sample. It is distinctly observed that plenty of Pt nanoparticles form on the perfect $Ni(OH)_2$. Scale bar, 50 nm.

Supplementary Table 2 | Structural parameters obtained from the EXAFS fitting analysis using β-$Ni(OH)_2$ as the reference compound (S_0^2 = 0.85).

Sample	Path	*N*	*R* (Å)	σ^2 (10^{-3}Å^2)	ΔE_0 (eV)
$Ni(OH)_x$ NBs	Ni-O	5.9 ± 0.5	2.06 ± 0.01	8.2 ± 1	-4.0 ± 0.1
	Ni-Ni	4.8 ± 0.5	3.12 ± 0.01	17 ± 2	-5.5 ± 0.3
perfect $Ni(OH)_2$	Ni-O	6.1 ± 0.5	2.06 ± 0.01	6.0 ± 1	-3.8 ± 0.1
	Ni-Ni	6.2 ± 0.5	3.12 ± 0.01	10.4 ± 2	-2.4 ± 0.1

Supplementary Methods

Computational methods. All calculations were performed by using density functional theory (DFT) implemented in the Vienna *Ab Initio* Simulation Package (VASP)[4,5]. The projected augmented wave (PAW) potential[6,7], and generalized gradient approximation of the Perdew-Burke-Ernzerhof functional[8,9], were employed to describe the electron-ion interaction and exchange-correlation energy, respectively. The DFT-D3 empirical correction method was employed to accurately describe the van der Waals interactions[10]. The energy cutoff for the plane-wave expansion was set to 400 eV. The energy convergence was set to 10^{-5} eV, and the residual force on each atom was smaller than 0.01 eV/Å for structural relaxations. Bulk nickel hydroxide ($Ni(OH)_2$) crystallizes are in a hexagonal structural with the $P\bar{3}m1$ space group. Our calculations show the lattice constant of 3.15 Å, which is in excellent agreement with the experimental value around 3.10 Å[11-13]. To investigate the adsorption of isolated Pt atoms on the surface of $Ni(OH)_2$, we use the monolayer $Ni(OH)_2$ with 4×4 supercell to avoid the interaction between the replicas due to the periodic boundary conditions. The vacuum distance normal to the slab was larger than 20 Å to eliminate spurious interactions between the periodically repeated images. The reciprocal space was sampled by gamma-centered *k* points in the Brillouin zone with a grid of 3×3×1.

In order to compare the stability of isolated Pt atoms adsorbed on different sites, the formation energy (E_f) was introduced and defined as:

$$E_f = E_{tot} - E_{sub} - E_{Pt} \quad \text{(Supplementary Equation 1)}$$

where E_{tot} is the total energy of substrate adsorbed with Pt atoms, E_{sub} is the energy of substrate without adsorbate, E_{Pt} is the energy of per Pt atom in the bulk structure.

The oxidation states of the Pt atoms adsorbed on the $Ni(OH)_2$ with or without Ni^{2+} vacancies are estimated by evaluating Bader charges of the atoms in the film and by normalizing them to Bader charges of bulk compounds[14]. The Bader charges of the Pt atom on the $Ni(OH)_2$ with and without Ni^{2+} vacancies are $1.25e$ and $0.77e$, respectively. When normalizing to bulk PtO_2 with Bader charge $1.41e$ and oxidation state of +4, the oxidation states of ISAS Pt species on the $Ni(OH)_2$ with or without Ni^{2+} vacancies are +3.55 and +2.70.

The catalytic mechanism of diboration reactions happening on single Pt atom has been proposed before[15-17]. As shown in Supplementary Fig. 12, a general cycle of Pt-catalyzed diboration reaction contains three steps as (1) oxidative addition of B-B bond to Pt, (2) insertion of C-C multiple bond and (3) reductive elimination of C-B bond from Pt.

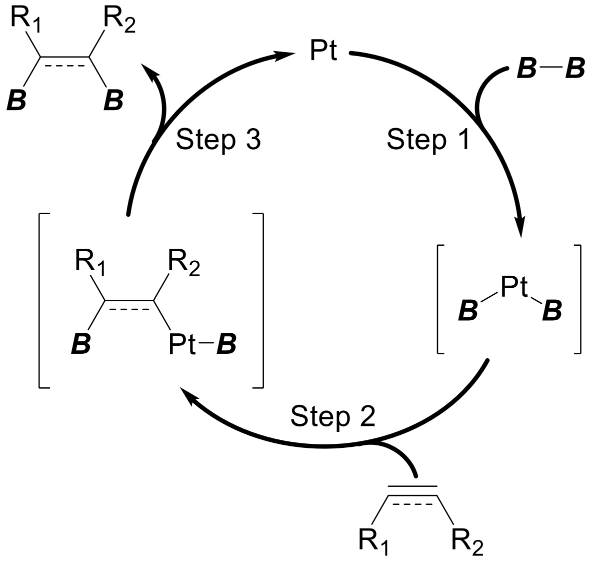

Supplementary Figure 12 | General cycle of Pt-catalyzed diboration reactions.

The diboration of phenylacetylene with bis(pinacolato)diboron (B_2pin_2) was used as the model reaction. According to the above reaction pathway, we calculated the energy evolution and reaction barrier of three crucial steps (Supplementary Fig. 13). The first step of diboration reaction contains the adsorption of B_2pin_2 and the dissociation of B-B bond, with an energy decrease of 3.49 eV. The energy barrier of dissociation of B-B bond is about 0.29 eV, which is easy to overcome in experiment. Therefore, the addition of B-B bond to isolated Pt atom is energetic and kinetic favorable in experiment. It is worth noting that not only the Ni^{2+} vacancies play an important role in locating isolated Pt atoms, but also the low-coordination oxygen atoms around the located Pt atoms benefit the dissociation of B-B bond. In the second step, the reaction process contains the adsorption of phenylacetylene and the insertion of Bpin on acetylenic bond, with an energy decrease of 1.41 eV. The barrier of insertion of Bpin on acetylenic bond is 1.57 eV, which is crucial process in the second step. The third step contains the insertion of another Bpin on ethylenic bond and the desorption of reacted chemical group, with an energy increase of 2.54 eV, indicating that it is an endothermic process. The barrier of insertion of another Bpin is 1.40 eV, which is consistent with that of the insertion of Bpin in the second step. To summarize, the overall diboration reaction of phenylacetylene with B_2pin_2 on $Pt_1/Ni(OH)_x$ is an exothermic reaction with an energy decrease of 2.36 eV and the insertion of the Bpin on acetylenic bond is the rate-limiting step for diboration reaction.

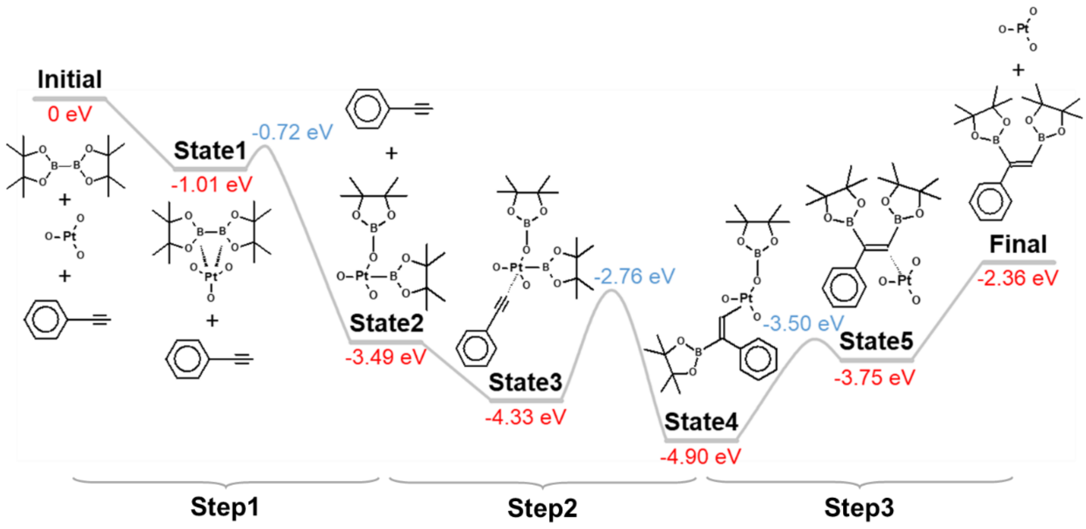

Supplementary Figure 13 | Energy evolution and reaction barrier of three proposed crucial steps of $Pt_1/Ni(OH)_x$ catalyzed diboration reactions.

Characterization of products. All products were isolated by flash column chromatography on silica gel with *n*-hexane/ethyl acetate as eluent. Among them, products **3aa-3da**, **3ga-3na** and **3ab** are known compounds and are characterized by comparison of their ^{1}H NMR and ^{13}C NMR spectroscopic data with those reported in the literature. Products **3ea** and **3fa** are new compounds and are characterized by ^{1}H NMR, ^{13}C NMR and high resolution mass (HRMS) spectra. All chemical shifts (δ) are reported in ppm and coupling constants (*J*) in Hz. All chemical shifts were reported relative to tetramethylsilane (0 ppm for ^{1}H), $CDCl_3$ (7.26 ppm for ^{1}H) and $CDCl_3$ (77.16 ppm for ^{13}C), respectively.

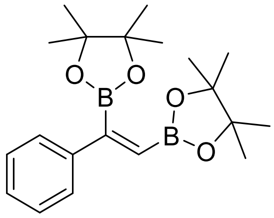

(*E*)-2,2'-(1-phenylethene-1,2-diyl)bis(4,4,5,5-tetramethyl-1,3,2-dioxaborolane) (3aa)[17]. ^{1}H NMR (400 MHz, $CDCl_3$): δ 7.45-7.42 (m, 2H), 7.32-7.28 (m, 2H), 7.25-7.21 (m, 1H), 6.29 (s, 1H), 1.37 (s, 12H), 1.30 (s, 12H); ^{13}C NMR (101 MHz, $CDCl_3$): δ 143.1, 128.3, 127.7, 126.6, 84.2, 83.6, 25.1, 24.9.

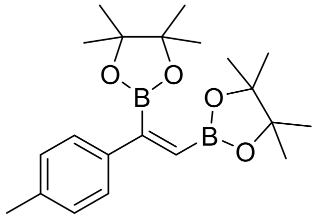

(*E*)-2,2'-(1-(p-tolyl)ethene-1,2-diyl)bis(4,4,5,5-tetramethyl-1,3,2-dioxaborolane) (3ba)[18]. ^{1}H NMR (400 MHz, $CDCl_3$): δ 7.35-7.32 (m, 2H), 7.12-7.10 (m, 2H), 6.26 (s, 1H), 2.31 (s, 3H), 1.37 (s, 12H), 1.30 (s, 12H); ^{13}C NMR (101 MHz, $CDCl_3$): δ 140.2, 137.5, 129.1, 126.5, 84.1, 83.6, 25.2, 24.9, 21.2.

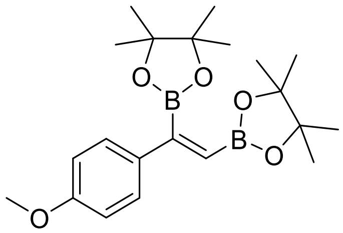

(*E*)-2,2'-(1-(4-methoxyphenyl)ethene-1,2-diyl)bis(4,4,5,5-tetramethyl-1,3,2-dioxaborolane) (3ca)[17]. ^{1}H NMR (400 MHz, $CDCl_3$): δ 7.41-7.38 (m, 2H), 6.86-6.82 (m, 2H), 6.22 (s, 1H), 3.78 (s, 3H), 1.38 (s, 12H), 1.30 (s, 12H); ^{13}C NMR (101 MHz, $CDCl_3$): δ 159.5, 135.5, 127.8, 113.8, 84.1, 83.5, 55.2, 25.1, 24.9.

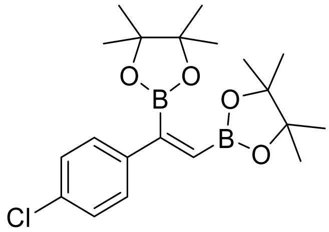

(*E*)-2,2'-(1-(4-chlorophenyl)ethene-1,2-diyl)bis(4,4,5,5-tetramethyl-1,3,2-dioxaborolane) (3da)[19]. ^{1}H NMR (400 MHz, $CDCl_3$): δ 7.38-7.35 (m, 2H), 7.28-7.25 (m, 2H), 6.27 (s, 1H), 1.37 (s, 12H), 1.31 (s, 12H); ^{13}C NMR (101 MHz, $CDCl_3$): δ 141.6, 133.6, 128.6, 128.0, 84.4, 83.8, 25.2, 25.0.

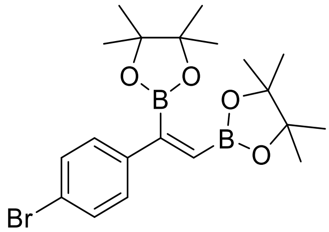

(*E*)-2,2'-(1-(4-bromophenyl)ethene-1,2-diyl)bis(4,4,5,5-tetramethyl-1,3,2-dioxaborolane) (3ea). ^{1}H NMR (400 MHz, $CDCl_3$): δ 7.44-7.41 (m, 2H), 7.32-7.28 (m, 2H), 6.28 (s, 1H), 1.36 (s, 12H), 1.30 (s, 12H); ^{13}C NMR (101 MHz, $CDCl_3$): δ 142.1, 131.5, 128.3, 121.8, 84.3, 83.8, 25.1, 25.0; HRMS (m/z): $[M+Na]^+$ calcd. for $C_{20}H_{29}B_2BrO_4Na$, 457.1333; found, 457.1323.

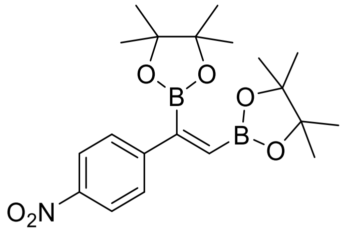

(*E*)-2,2'-(1-(4-nitrophenyl)ethene-1,2-diyl)bis(4,4,5,5-tetramethyl-1,3,2-dioxaborolane) (3fa). ^{1}H NMR (400 MHz, $CDCl_3$): δ 8.18-8.15 (m, 2H), 7.57-7.54 (m, 2H), 6.41 (s, 1H), 1.37 (s, 12H), 1.32 (s, 12H); ^{13}C NMR (101 MHz, $CDCl_3$): δ 150.0, 147.2, 127.6, 123.8, 84.7, 84.2, 25.1, 25.0. HRMS (m/z): $[M+Na]^+$ calcd. for $C_{20}H_{29}B_2NO_6Na$, 424.2079; found, 424.2070.

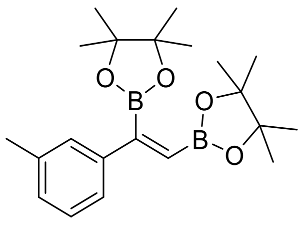

(*E*)-2,2'-(1-(m-tolyl)ethene-1,2-diyl)bis(4,4,5,5-tetramethyl-1,3,2-dioxaborolane) (3ga)[19]. ^{1}H NMR (400 MHz, $CDCl_3$): δ 7.26-7.22 (m, 2H), 7.20-7.16 (m, 1H), 7.07-7.04 (m, 1H), 6.27 (s, 1H), 2.32 (s, 3H), 1.37 (s, 12H), 1.30 (s, 12H); ^{13}C NMR (101 MHz, $CDCl_3$): δ 143.1, 137.8, 128.4, 128.2, 127.4, 123.7, 84.1, 83.6, 25.1, 24.9, 21.5.

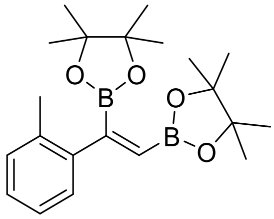

(*E*)-2,2'-(1-(o-tolyl)ethene-1,2-diyl)bis(4,4,5,5-tetramethyl-1,3,2-dioxaborolane) (3ha)[17]. ^{1}H NMR (400 MHz, $CDCl_3$): δ 7.12 (s, 4H), 6.02 (s, 1H), 2.31 (s, 3H), 1.32 (s, 12H), 1.29 (s, 12H); ^{13}C NMR (101 MHz, $CDCl_3$): δ 144.7, 134.6, 130.0, 128.1, 126.8, 125.7, 84.0, 83.7, 25.0(4), 24.9(6), 20.6.

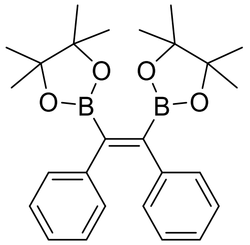

(*Z*)-1,2-diphenyl-1,2-bis(4,4,5,5-tetramethyl-1,3,2-dioxaborolan-2-yl)ethane (3ia)[17]. ^{1}H NMR (400 MHz, $CDCl_3$): δ 7.09-7.02 (m, 6H), 6.96-6.93 (m, 4H), 1.32 (s, 24H); ^{13}C NMR (101 MHz, $CDCl_3$): δ 141.4, 129.4, 127.5, 125.9, 84.2, 25.0.

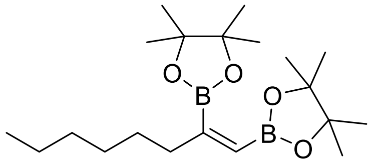

(*E*)-2,2'-(oct-1-ene-1,2-diyl)bis(4,4,5,5-tetramethyl-1,3,2-dioxaborolane) (3ja)[17]. ^{1}H NMR (400 MHz, $CDCl_3$): δ 5.84 (s, 1H), 2.21 (t, J = 7.6 Hz, 2H), 1.43-1.39 (m, 2H), 1.31-1.26 (m, 30H), 0.88-0.85 (m, 3H); ^{13}C NMR (101 MHz, $CDCl_3$): δ 83.7, 83.3, 40.0, 31.9, 29.2, 28.7, 25.0, 25.0, 24.9, 22.7, 14.2.

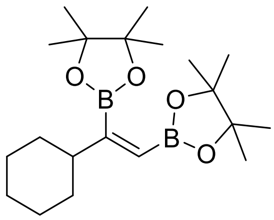

(*E*)-2,2'-(1-cyclohexylethene-1,2-diyl)bis(4,4,5,5-tetramethyl-1,3,2-dioxaborolane) (3ka)[18]. ^{1}H NMR (400 MHz, $CDCl_3$): δ 5.79 (s, 1H), 2.12-2.05 (m, 1H), 1.75-1.70 (m, 4H), 1.32 (s, 12H), 1.25 (s, 12H), 1.22-1.08 (m, 6H); ^{13}C NMR (101 MHz, $CDCl_3$): δ 83.7, 83.3, 47.8, 32.4, 26.7, 26.4, 25.2, 25.0.

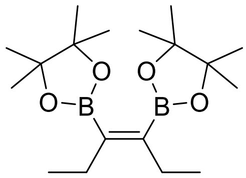

(*Z*)-2,2'-(hex-3-ene-3,4-diyl)bis(4,4,5,5-tetramethyl-1,3,2-dioxaborolane) (3la)[20]. ^{1}H NMR (400 MHz, $CDCl_3$): δ 2.20 (q, *J* = 7.6 Hz, 4H), 1.28 (s, 24H), 0.96 (t, *J* = 7.6 Hz, 6H); ^{13}C NMR (101 MHz, $CDCl_3$): δ 83.4, 25.0, 23.7, 14.4.

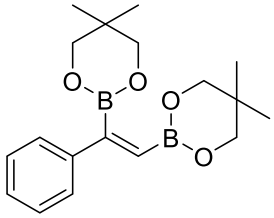

(*E*)-2,2'-(1-phenylethene-1,2-diyl)bis(5,5-dimethyl-1,3,2-dioxaborinane) (3ab)[21]. ^{1}H NMR (400 MHz, $CDCl_3$): δ 7.46-7.43 (m, 2H), 7.31-7.27 (m, 2H), 7.24-7.20 (m, 1H), 6.17 (s, 1H), 3.72 (s, 4H), 3.69 (s, 4H), 1.06 (s, 6H), 1.01 (s, 6H); ^{13}C NMR (101 MHz, $CDCl_3$): δ 143.2, 128.4, 127.4, 126.7, 72.6, 72.5, 32.0, 31.8, 22.4, 22.1.

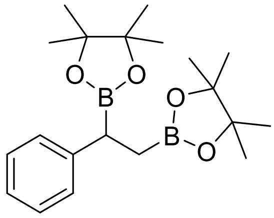

2,2'-(1-phenylethane-1,2-diyl)bis(4,4,5,5-tetramethyl-1,3,2-dioxaborolane) (3ma)[22]. ^{1}H NMR (400 MHz, $CDCl_3$): δ 7.23-7.22 (m, 4H), 7.12-7.07 (m, 1H), 2.52 (dd, J_1 = 11.0 Hz, J_2 = 5.6 Hz, 1H), 1.38-1.36 (m, 1H), 1.20 (s, 12H), 1.19 (s, 6H), 1.17 (s, 6H), 1.11 (dd, J_1 = 16.0 Hz, J_2 = 5.7 Hz, 1H); ^{13}C NMR (101 MHz, $CDCl_3$): δ 145.5, 128.3, 128.0, 125.0, 83.3, 83.1, 25.1, 24.8(3), 24.8(0), 24.6.

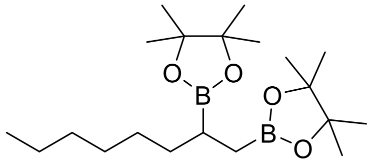

2,2'-(octane-1,2-diyl)bis(4,4,5,5-tetramethyl-1,3,2-dioxaborolane) (3na)[22]. ^{1}H NMR (400 MHz, $CDCl_3$): δ 1.46-1.41 (m, 1H), 1.28-1.25 (m, 8H), 1.24 (s, 12H), 1.23 (s, 12H), 1.15-1.07 (m, 2H), 0.88-0.80 (m, 5H); ^{13}C NMR (101 MHz, $CDCl_3$): δ 82.9, 82.8, 34.0, 32.0, 30.0, 29.0, 25.0(2), 24.9(6), 24.8(9), 24.8(6), 22.8, 14.2.

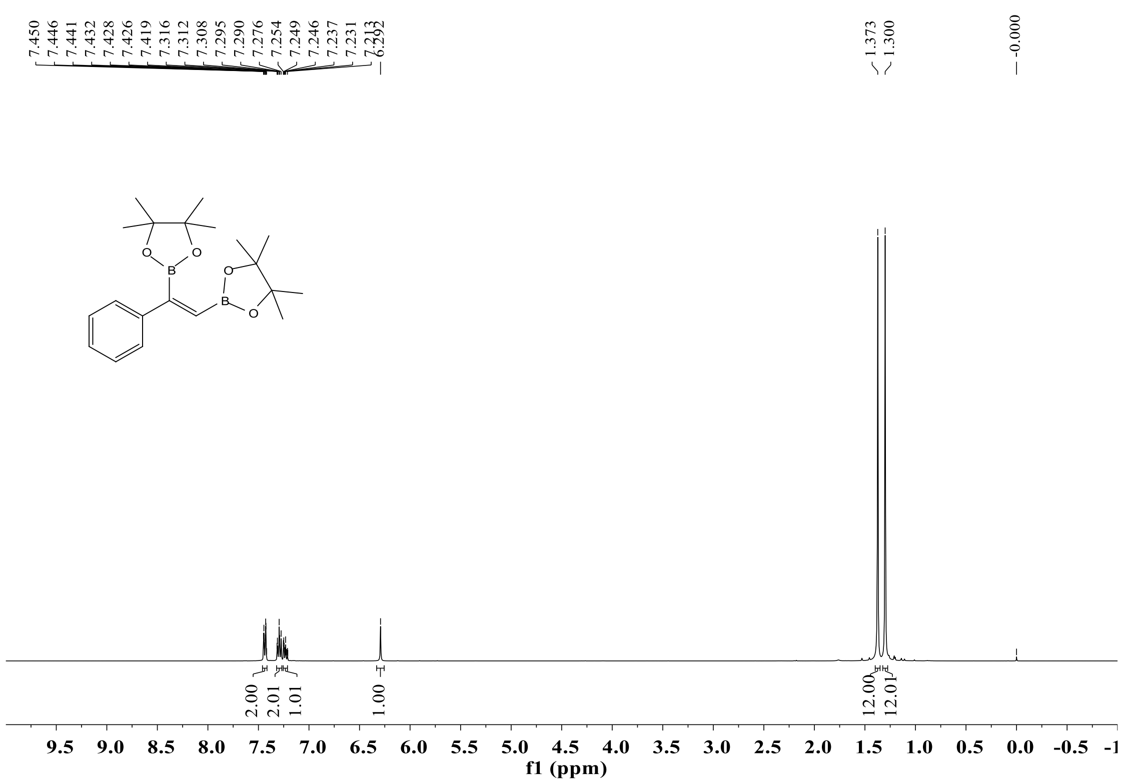

Supplementary Figure 14 | ^{1}H NMR (400 MHz, $CDCl_3$) spectrum of (*E*)-2,2'-(1-phenylethene-1,2-diyl)bis(4,4,5,5-tetramethyl-1,3,2-dioxaborolane) (3aa).

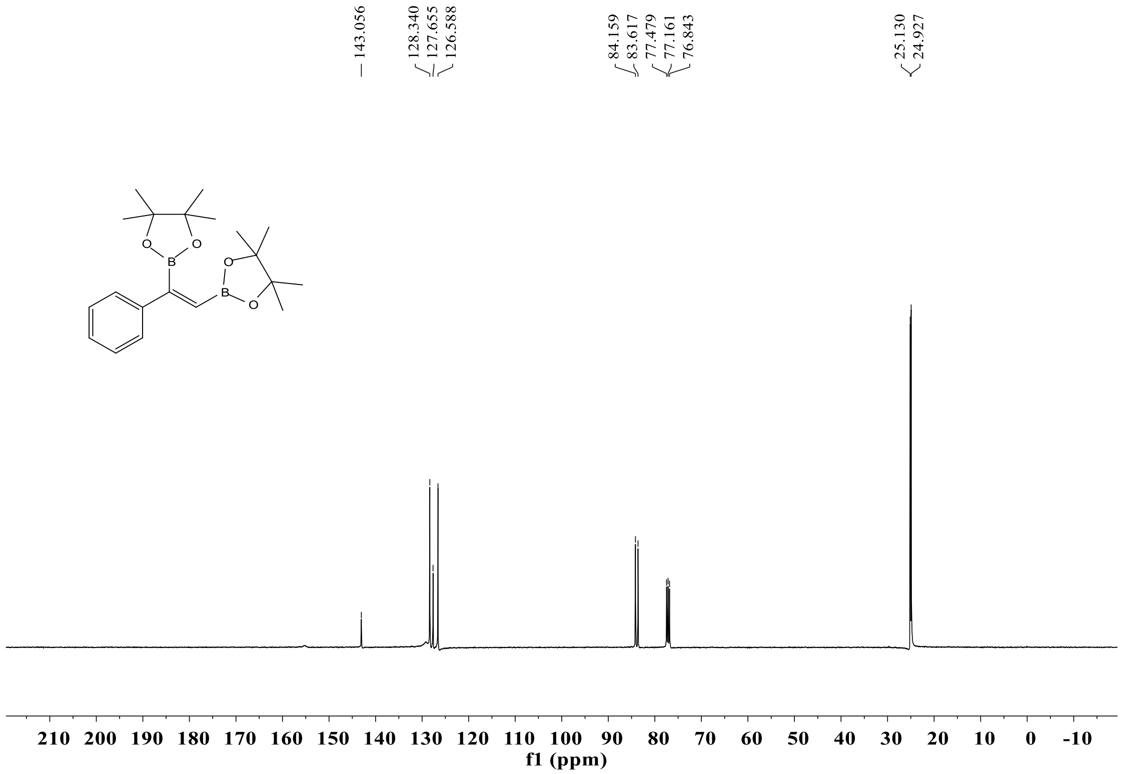

Supplementary Figure 15 | ^{13}C NMR (101 MHz, $CDCl_3$) spectrum of (*E*)-2,2'-(1-phenylethene-1,2-diyl)bis(4,4,5,5-tetramethyl-1,3,2-dioxaborolane) (3aa).

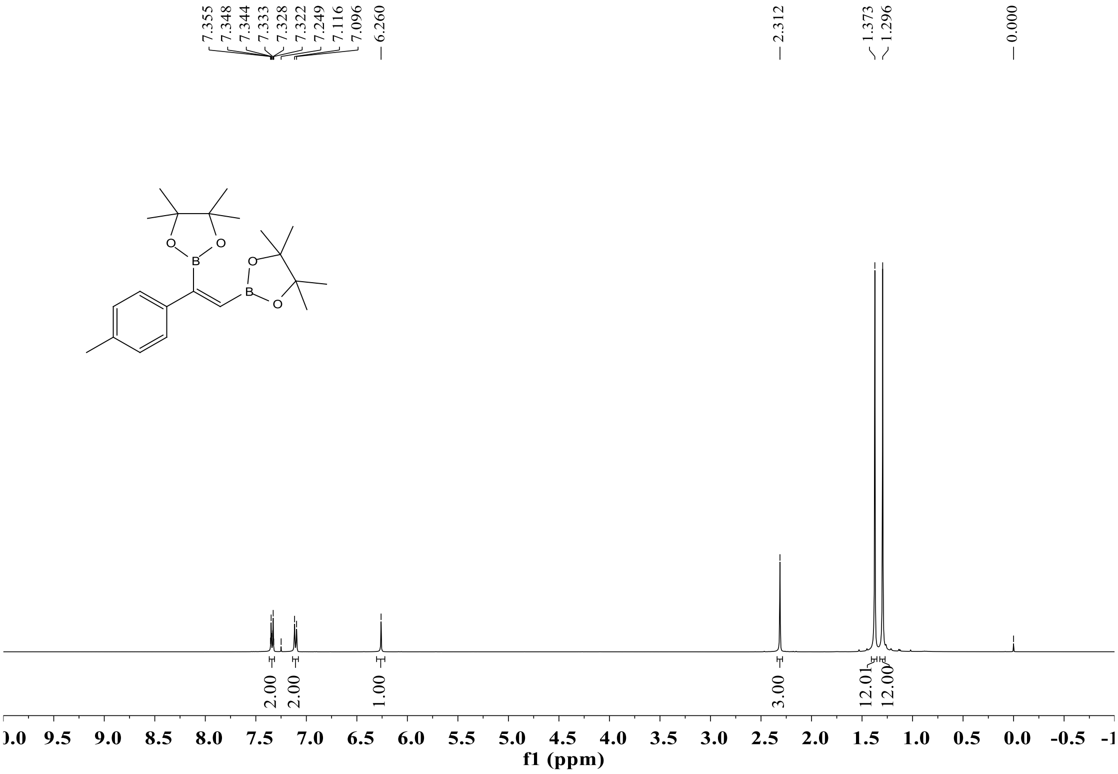

Supplementary Figure 16 | ^{1}H NMR (400 MHz, $CDCl_3$) spectrum of (*E*)-2,2'-(1-(p-tolyl)ethene-1,2-diyl)bis(4,4,5,5-tetramethyl-1,3,2-dioxaborolane) (3ba)

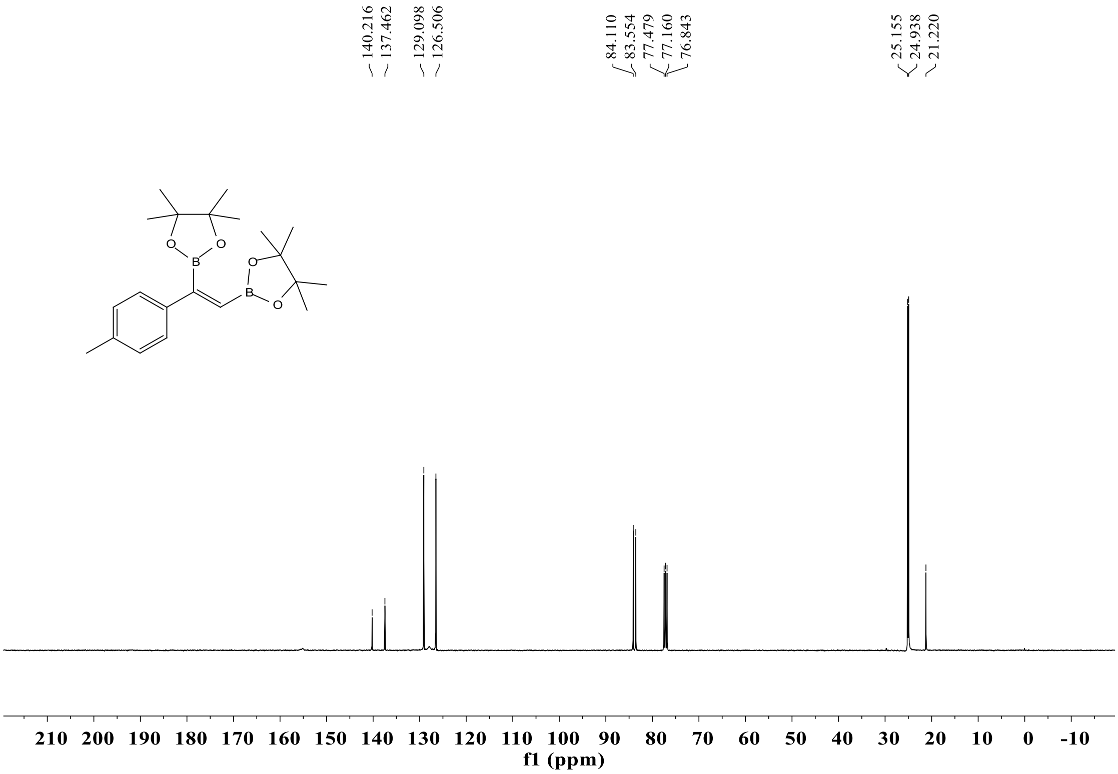

Supplementary Figure 17 | ^{13}C NMR (101 MHz, $CDCl_3$) spectrum of (*E*)-2,2'-(1-(p-tolyl)ethene-1,2-diyl)bis(4,4,5,5-tetramethyl-1,3,2-dioxaborolane) (3ba)

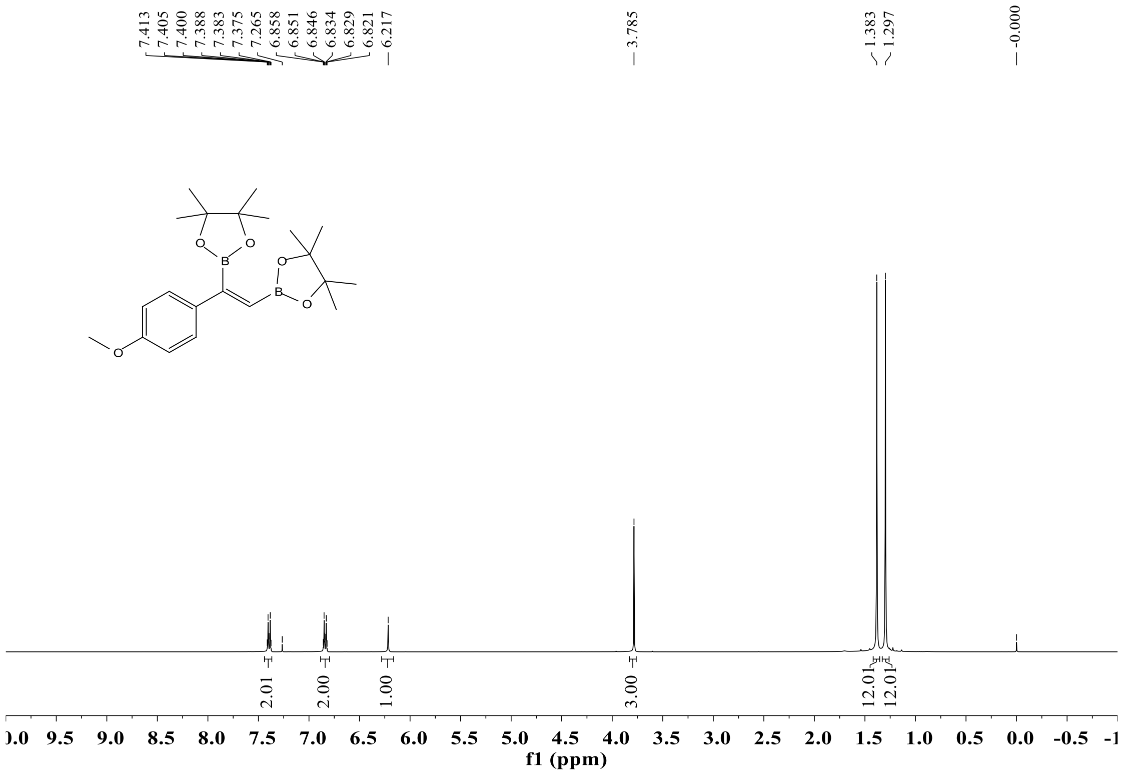

Supplementary Figure 18 | ^{1}H NMR (400 MHz, $CDCl_3$) spectrum of (*E*)-2,2'-(1-(4-methoxyphenyl)ethene-1,2-diyl)bis(4,4,5,5-tetramethyl-1,3,2-dioxaborolane) (3ca)

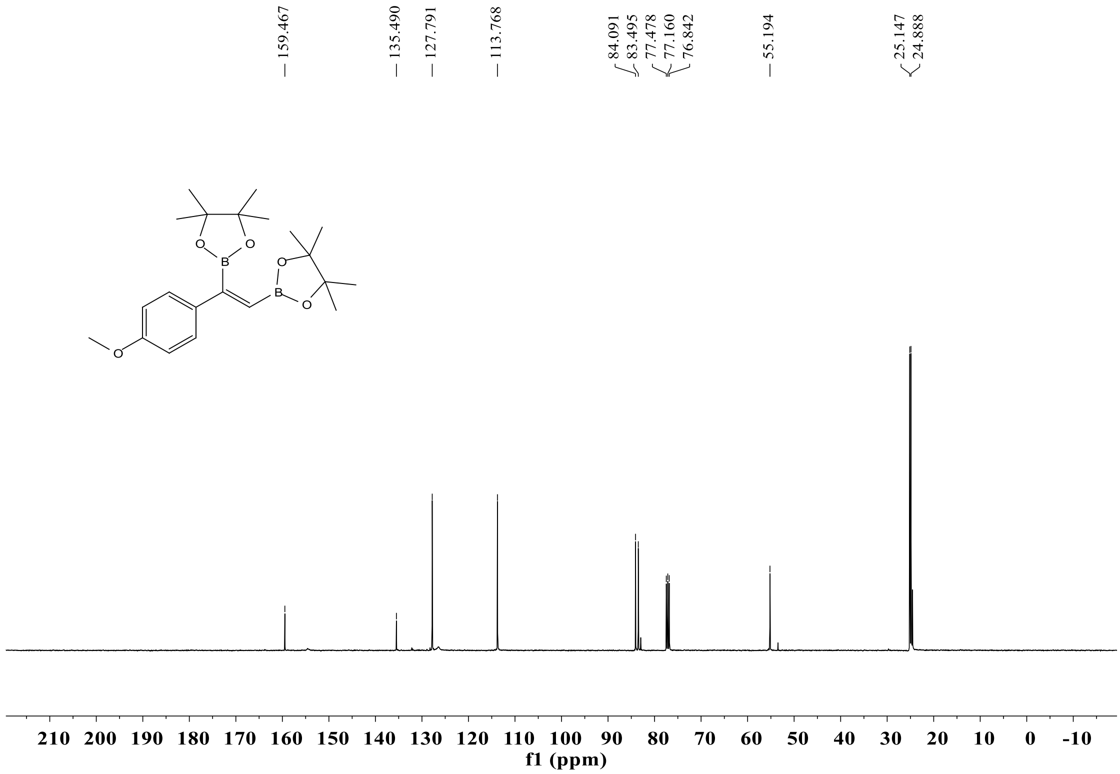

Supplementary Figure 19 | ^{13}C NMR (101 MHz, $CDCl_3$) spectrum of (*E*)-2,2'-(1-(4-methoxyphenyl)ethene-1,2-diyl)bis(4,4,5,5-tetramethyl-1,3,2-dioxaborolane) (3ca)

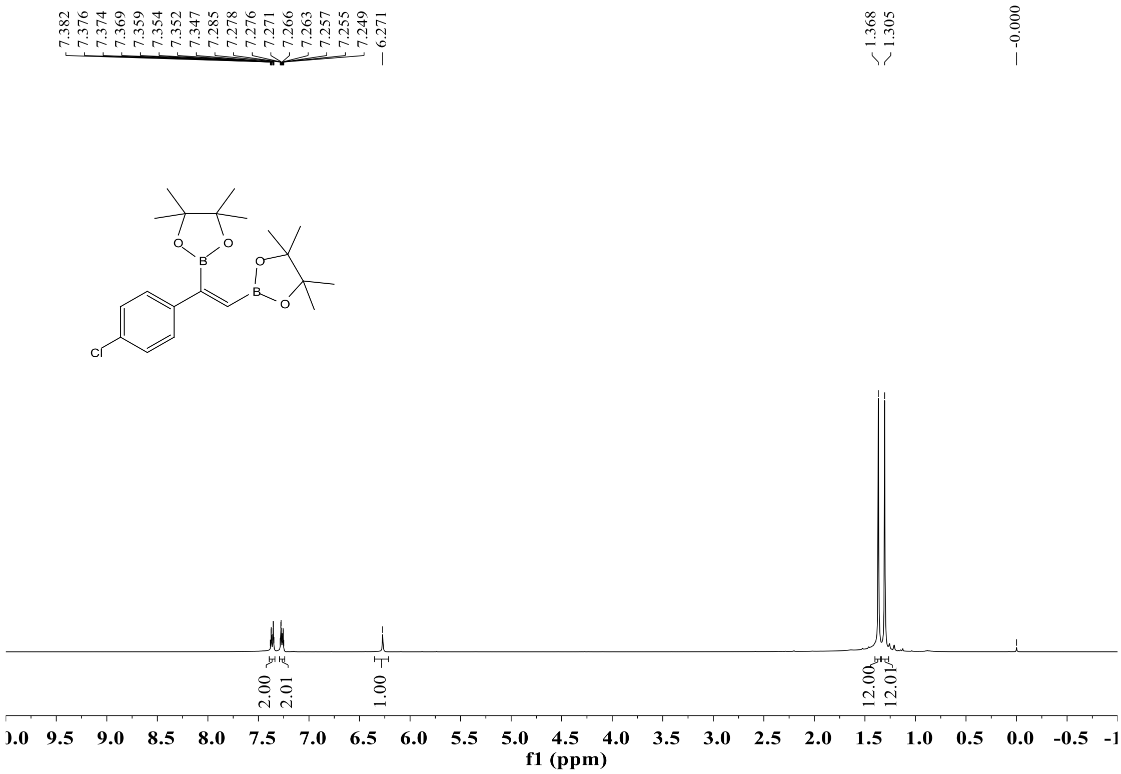

Supplementary Figure 20 | ^{1}H NMR (400 MHz, $CDCl_3$) spectrum of (*E*)-2,2'-(1-(4-chlorophenyl)ethene-1,2-diyl)bis(4,4,5,5-tetramethyl-1,3,2-dioxaborolane) (3da)

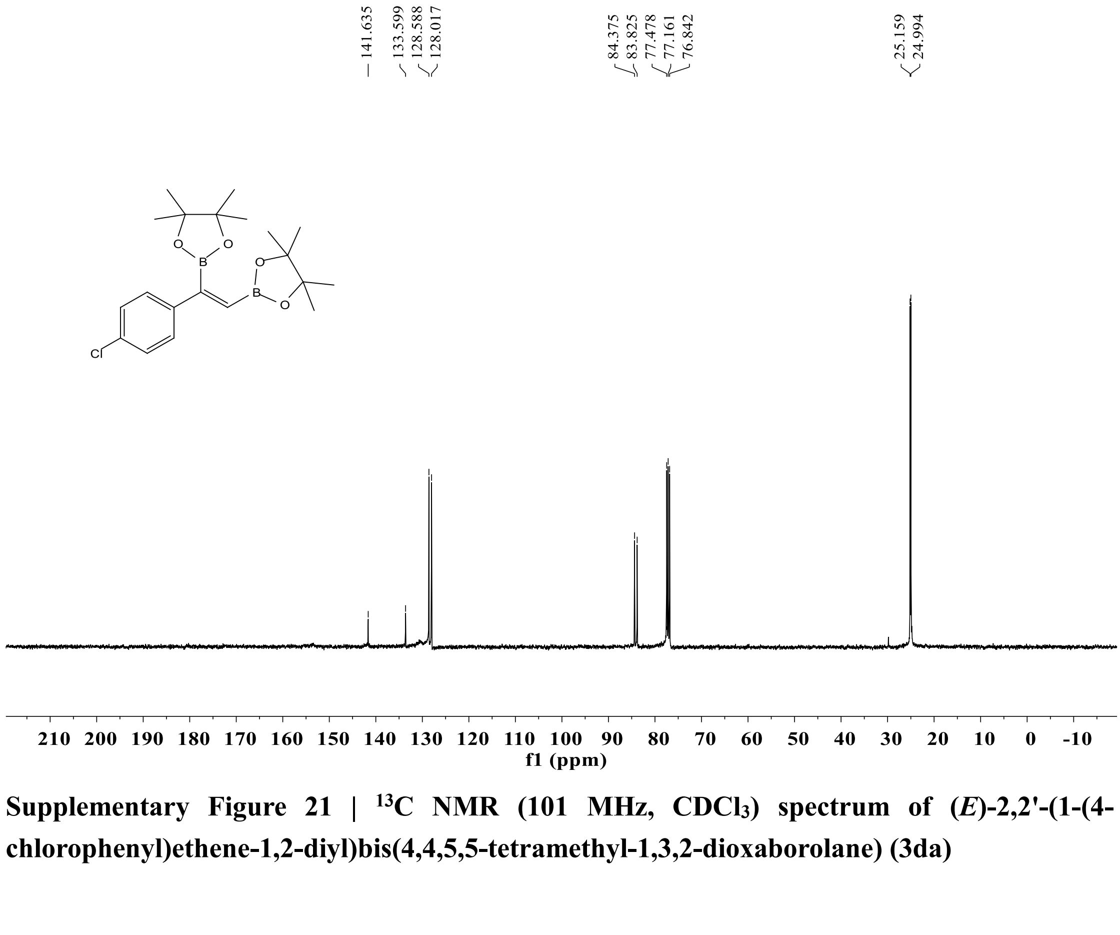

Supplementary Figure 21 | ^{13}C NMR (101 MHz, $CDCl_3$) spectrum of (*E*)-2,2'-(1-(4-chlorophenyl)ethene-1,2-diyl)bis(4,4,5,5-tetramethyl-1,3,2-dioxaborolane) (3da)

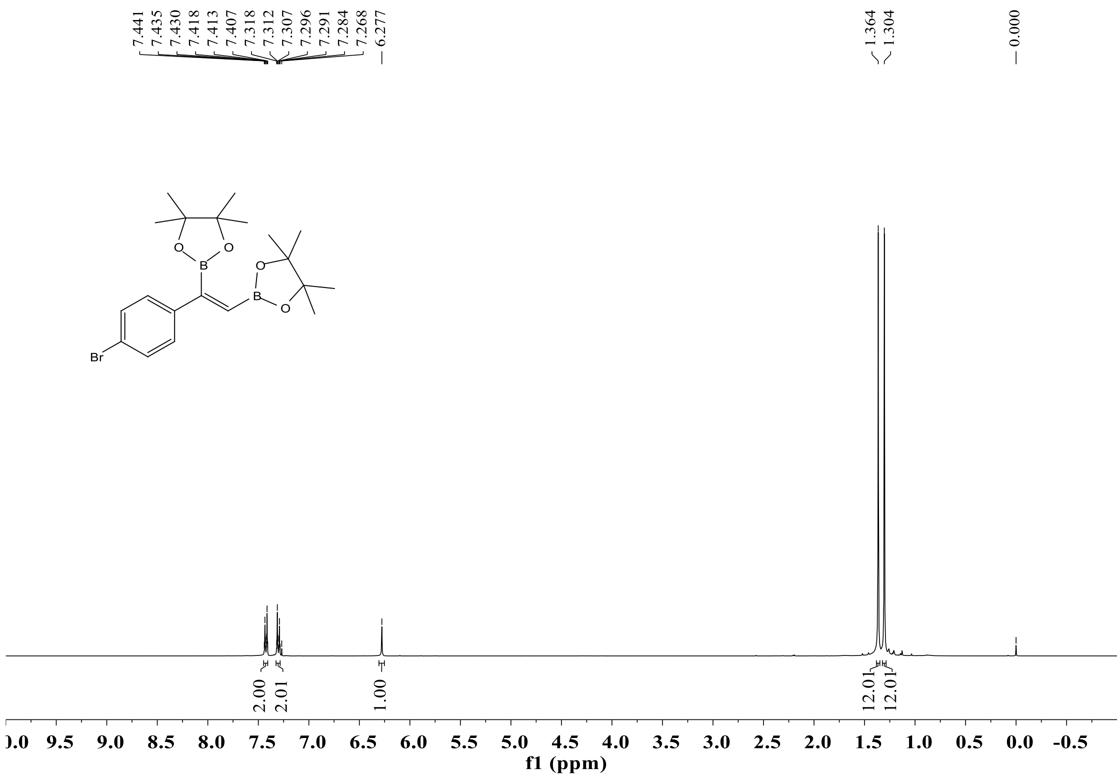

Supplementary Figure 22 | ^{1}H NMR (400 MHz, $CDCl_3$) spectrum of (*E*)-2,2'-(1-(4-bromophenyl)ethene-1,2-diyl)bis(4,4,5,5-tetramethyl-1,3,2-dioxaborolane) (3ea)

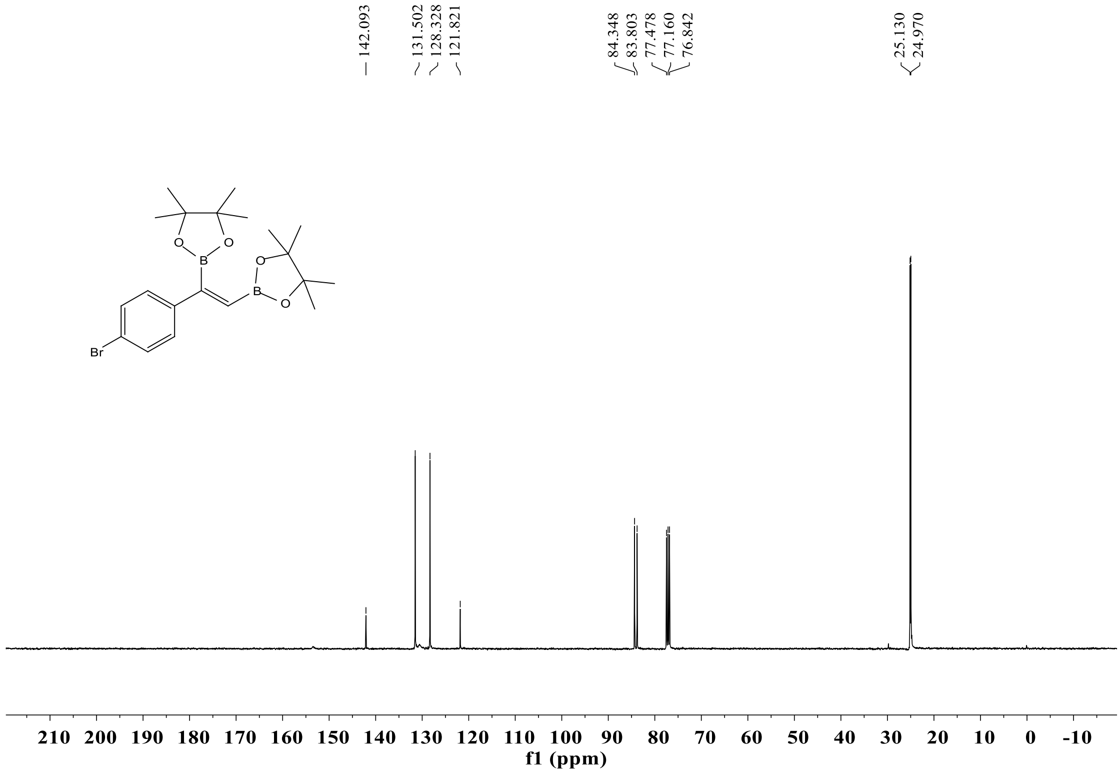

Supplementary Figure 23 | ^{13}C NMR (101 MHz, $CDCl_3$) spectrum of (*E*)-2,2'-(1-(4-bromophenyl)ethene-1,2-diyl)bis(4,4,5,5-tetramethyl-1,3,2-dioxaborolane) (3ea)

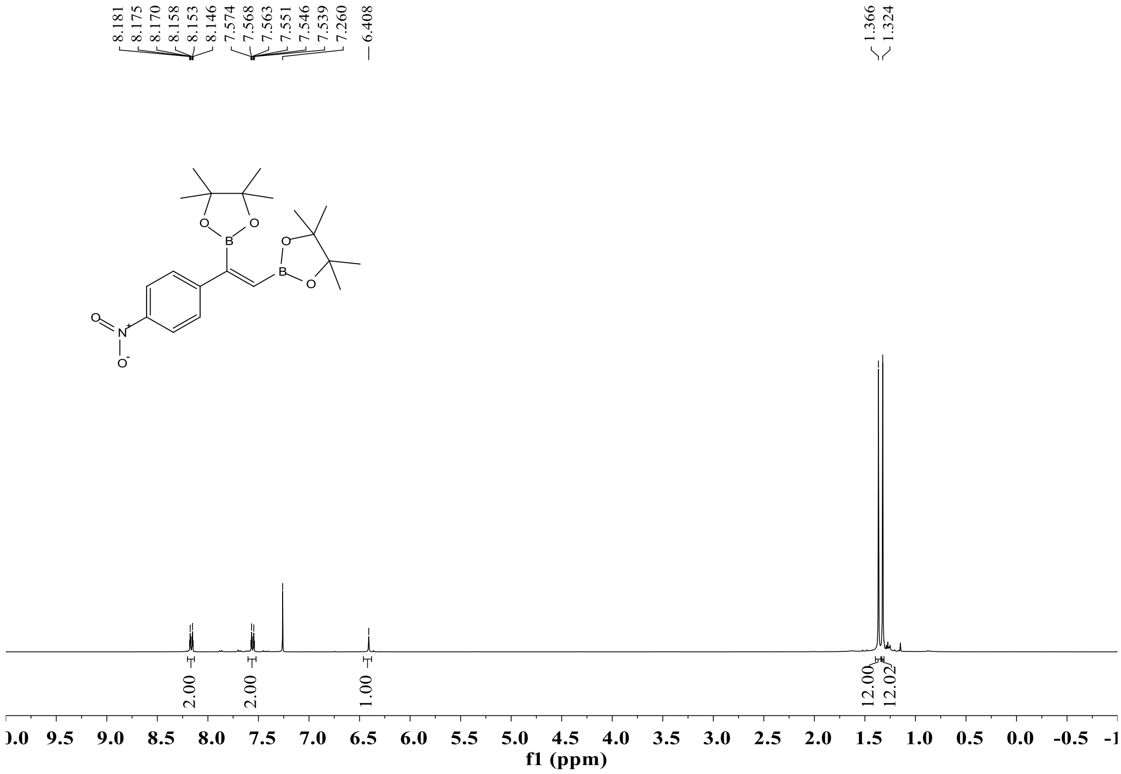

Supplementary Figure 24 | ^{1}H NMR (400 MHz, $CDCl_3$) spectrum of (*E*)-2,2'-(1-(4-nitrophenyl)ethene-1,2-diyl)bis(4,4,5,5-tetramethyl-1,3,2-dioxaborolane) (3fa)

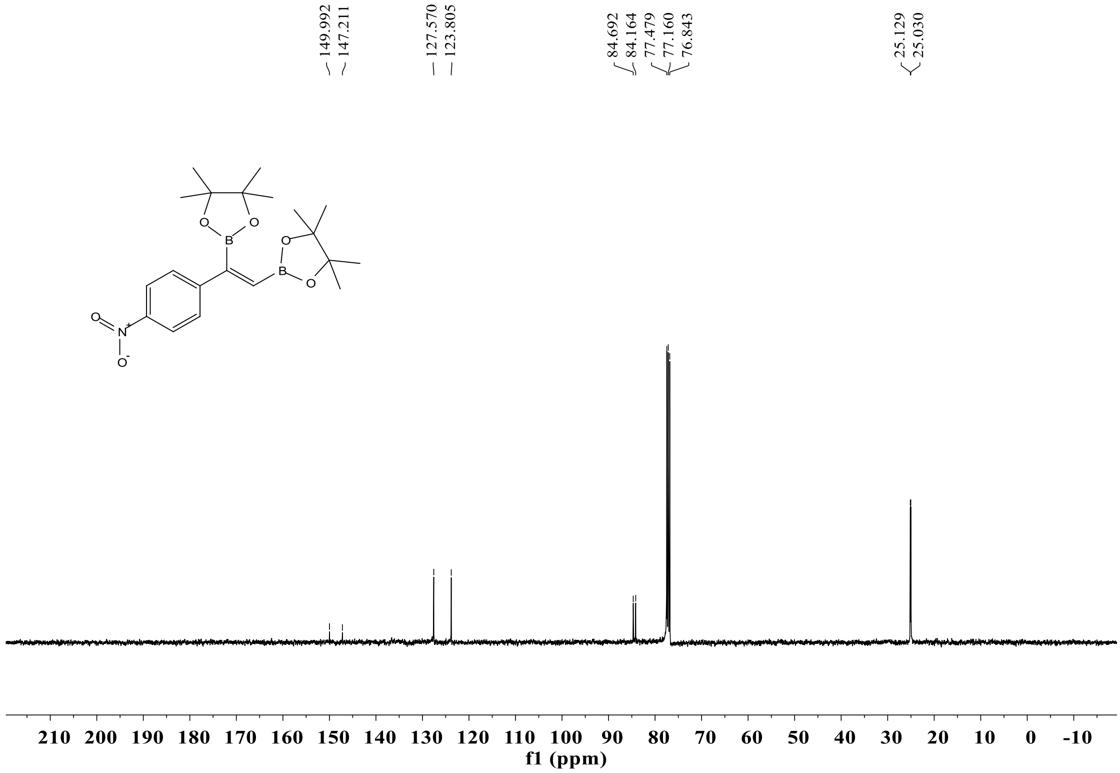

Supplementary Figure 25 | ^{13}C NMR (101 MHz, $CDCl_3$) spectrum of (*E*)-2,2'-(1-(4-nitrophenyl)ethene-1,2-diyl)bis(4,4,5,5-tetramethyl-1,3,2-dioxaborolane) (3fa)

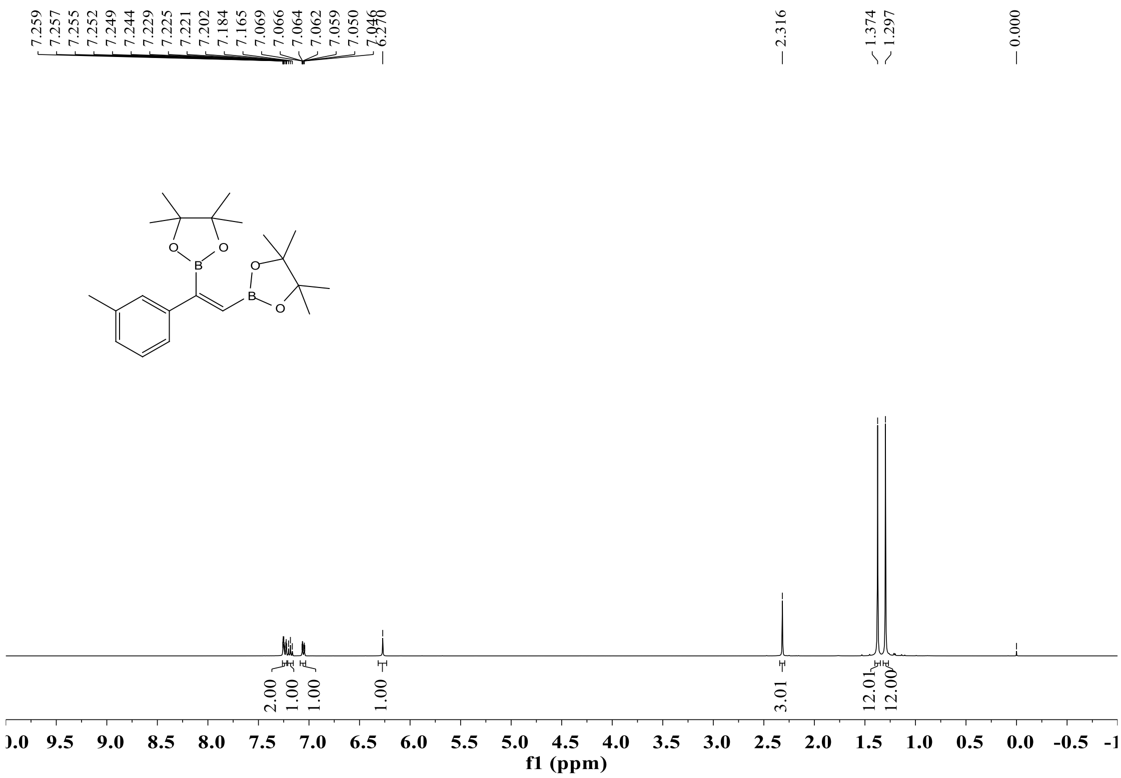

Supplementary Figure 26 | ^{1}H NMR (400 MHz, $CDCl_3$) spectrum of (*E*)-2,2'-(1-(m-tolyl)ethene-1,2-diyl)bis(4,4,5,5-tetramethyl-1,3,2-dioxaborolane) (3ga)

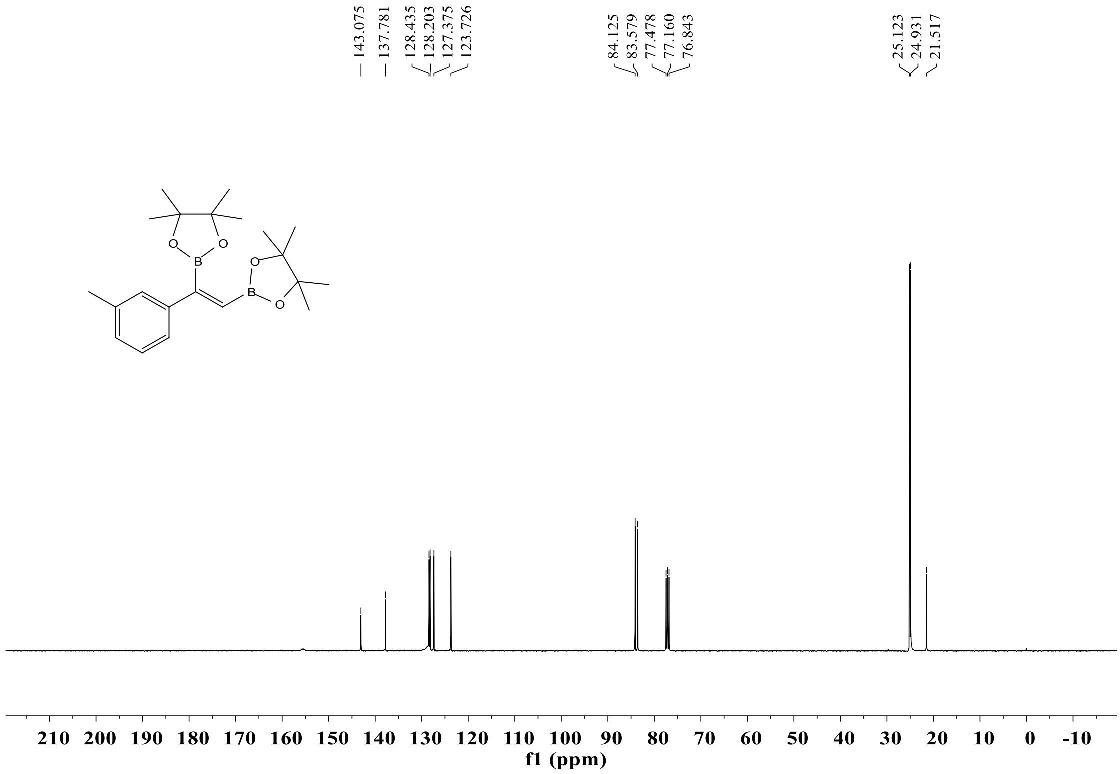

Supplementary Figure 27 | ^{13}C NMR (101 MHz, $CDCl_3$) spectrum of (*E*)-2,2'-(1-(m-tolyl)ethene-1,2-diyl)bis(4,4,5,5-tetramethyl-1,3,2-dioxaborolane) (3ga)

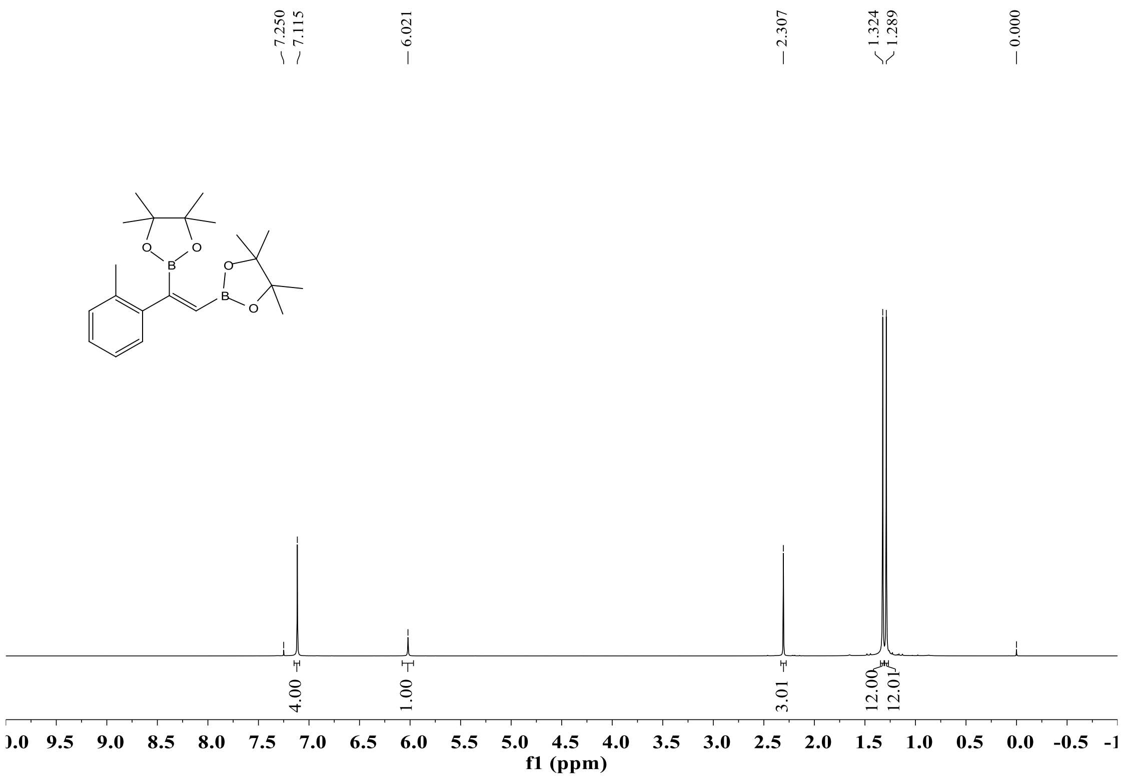

Supplementary Figure 28 | ^{1}H NMR (400 MHz, $CDCl_3$) spectrum of (*E*)-2,2'-(1-(o-tolyl)ethene-1,2-diyl)bis(4,4,5,5-tetramethyl-1,3,2-dioxaborolane) (3ha)

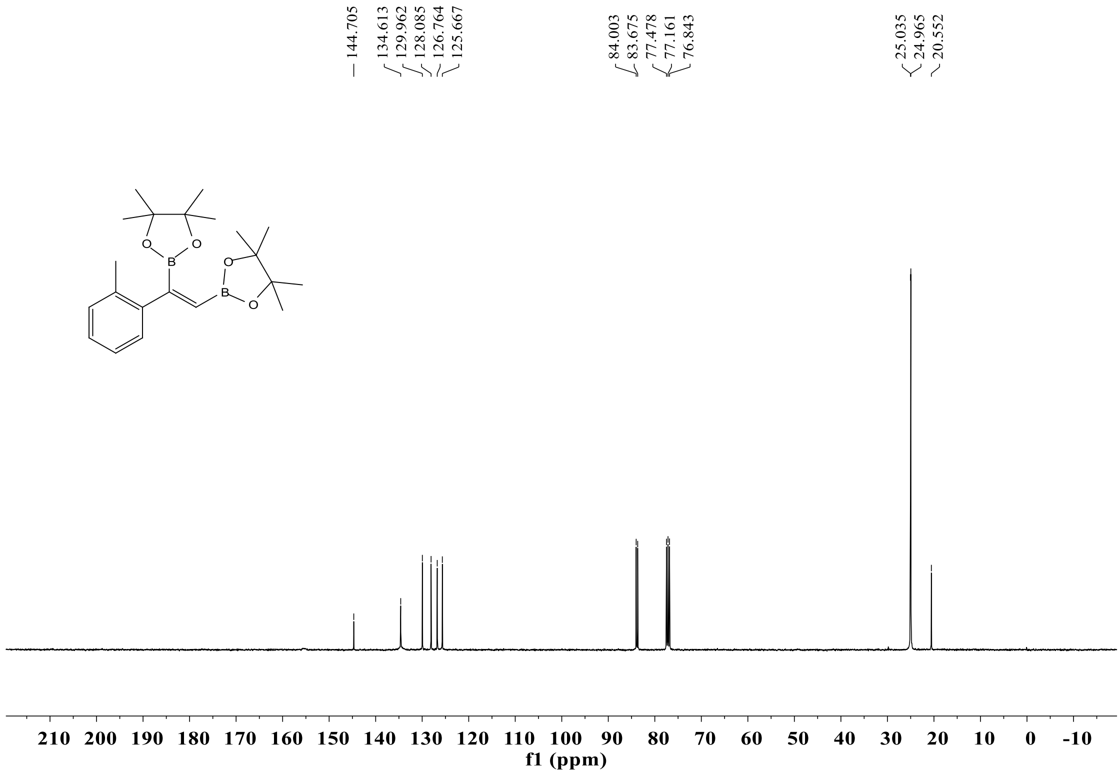

Supplementary Figure 29 | ^{13}C NMR (101 MHz, $CDCl_3$) spectrum of (*E*)-2,2'-(1-(o-tolyl)ethene-1,2-diyl)bis(4,4,5,5-tetramethyl-1,3,2-dioxaborolane) (3ha)

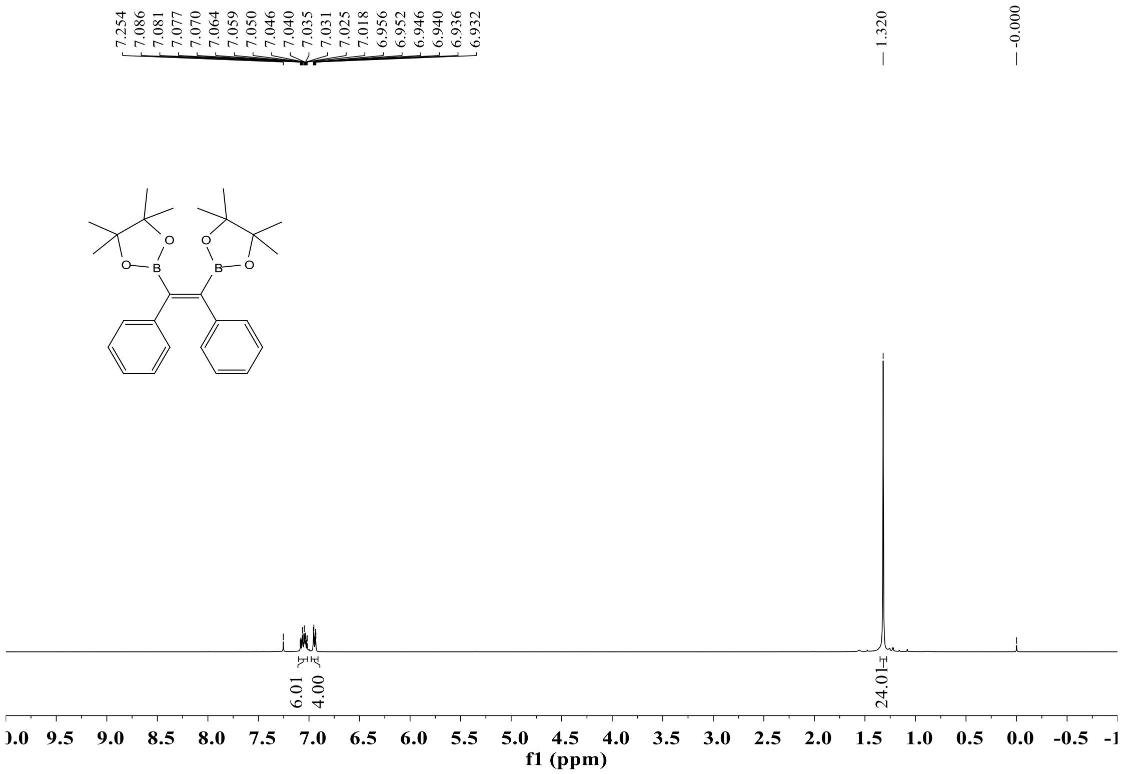

Supplementary Figure 30 | ^{1}H NMR (400 MHz, $CDCl_3$) spectrum of (*Z*)-1,2-diphenyl-1,2-bis(4,4,5,5-tetramethyl-1,3,2-dioxaborolan-2-yl)ethane (3ia)

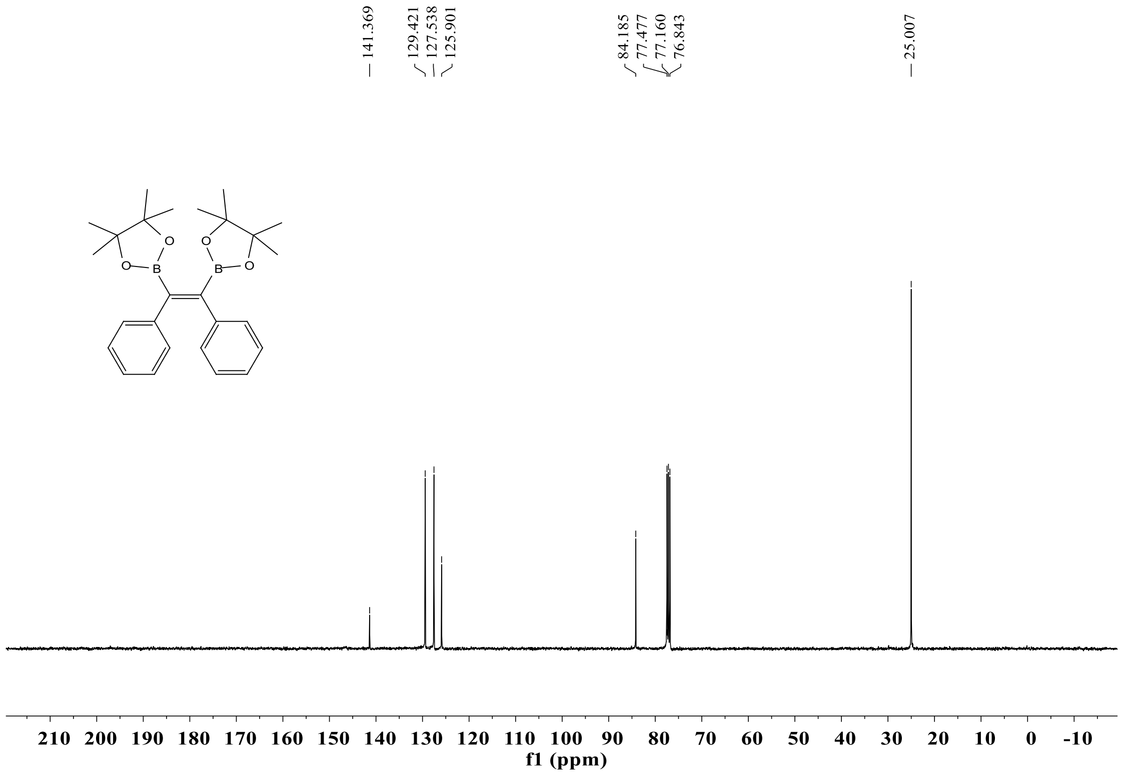

Supplementary Figure 31 | ^{13}C NMR (101 MHz, $CDCl_3$) spectrum of (*Z*)-1,2-diphenyl-1,2-bis(4,4,5,5-tetramethyl-1,3,2-dioxaborolan-2-yl)ethane (3ia)

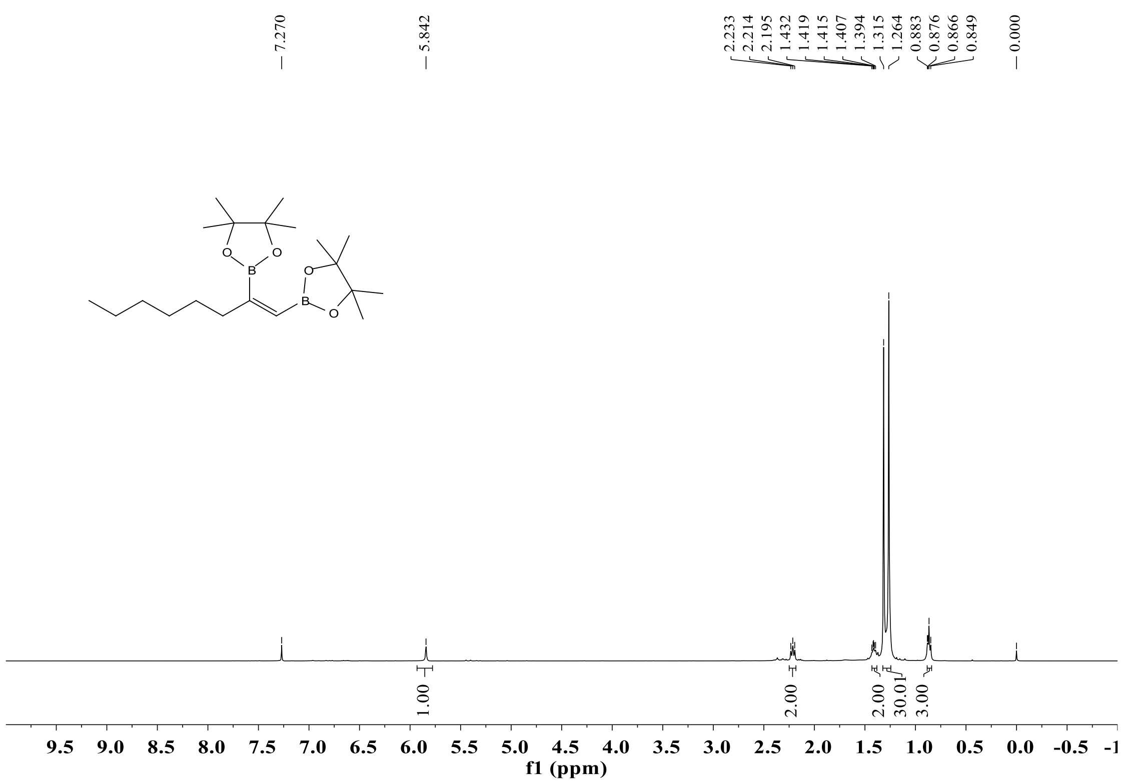

Supplementary Figure 32 | ^{1}H NMR (400 MHz, $CDCl_3$) spectrum of (*E*)-2,2'-(oct-1-ene-1,2-diyl)bis(4,4,5,5-tetramethyl-1,3,2-dioxaborolane) (3ja)

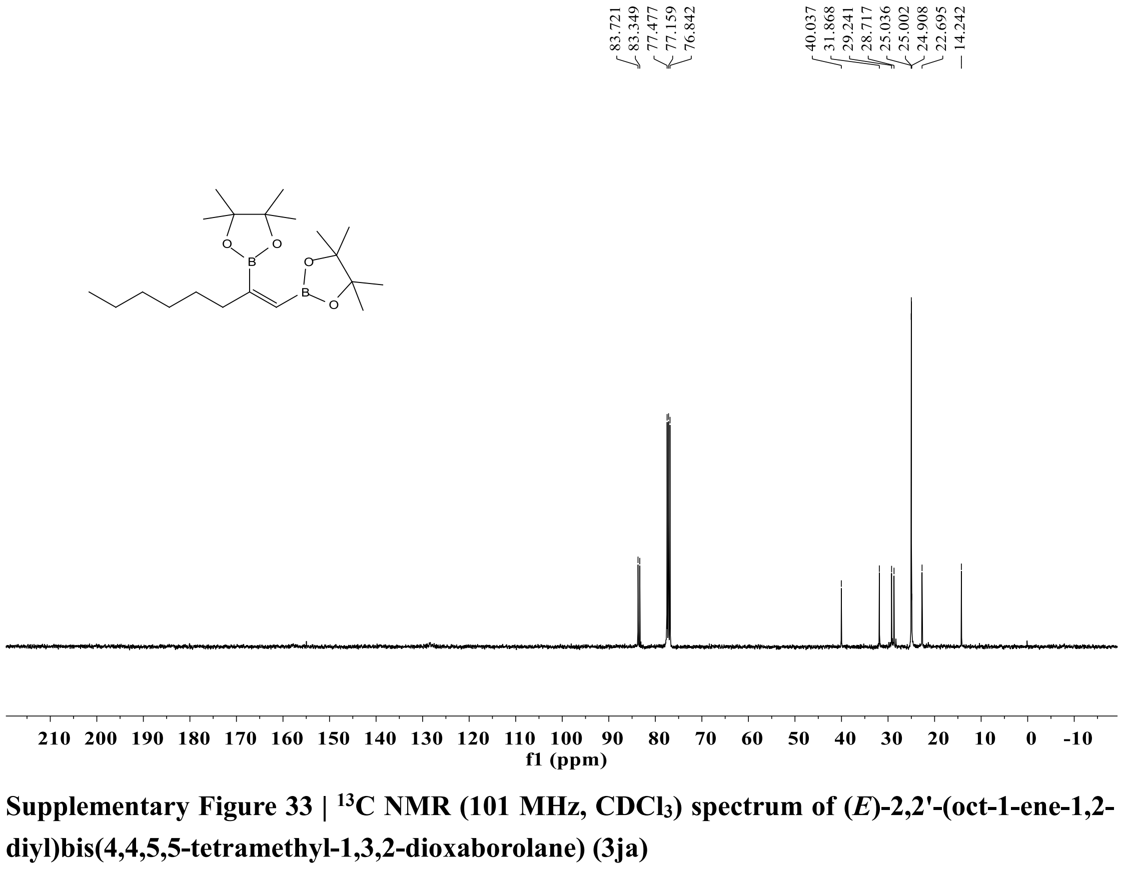

Supplementary Figure 33 | ^{13}C NMR (101 MHz, $CDCl_3$) spectrum of (*E*)-2,2'-(oct-1-ene-1,2-diyl)bis(4,4,5,5-tetramethyl-1,3,2-dioxaborolane) (3ja)

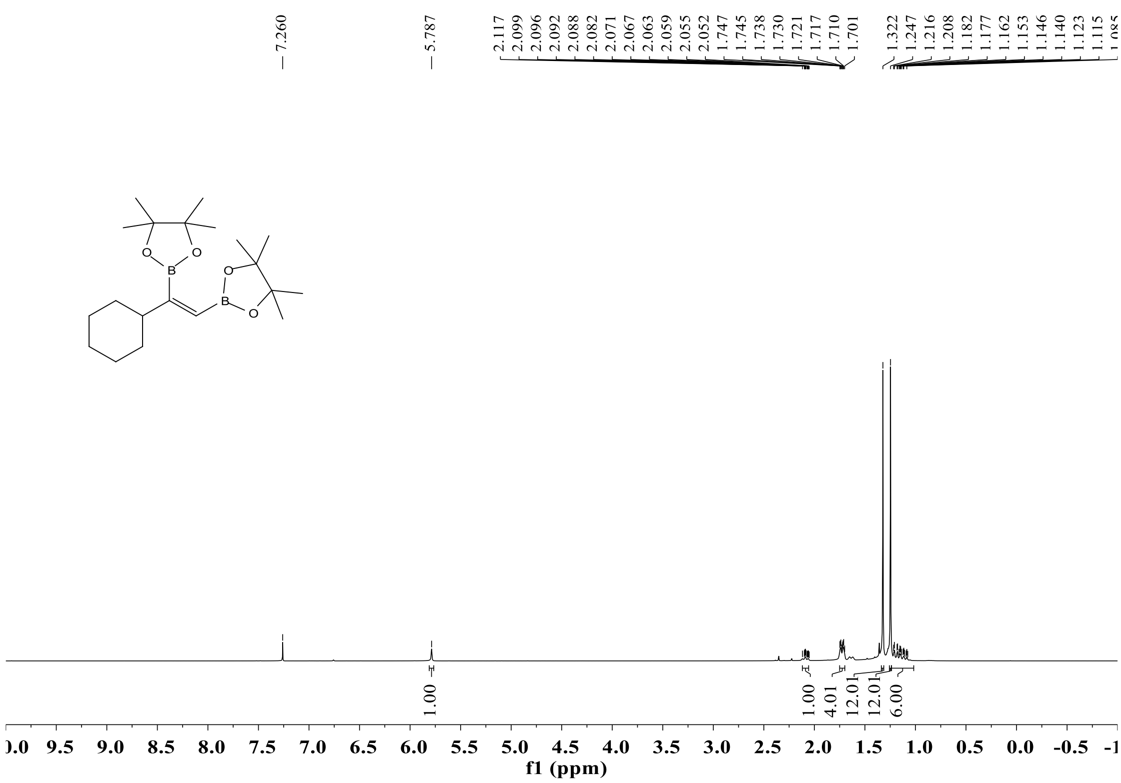

Supplementary Figure 34 | ^{1}H NMR (400 MHz, $CDCl_3$) spectrum of (*E*)-2,2'-(1-cyclohexylethene-1,2-diyl)bis(4,4,5,5-tetramethyl-1,3,2-dioxaborolane) (3ka)

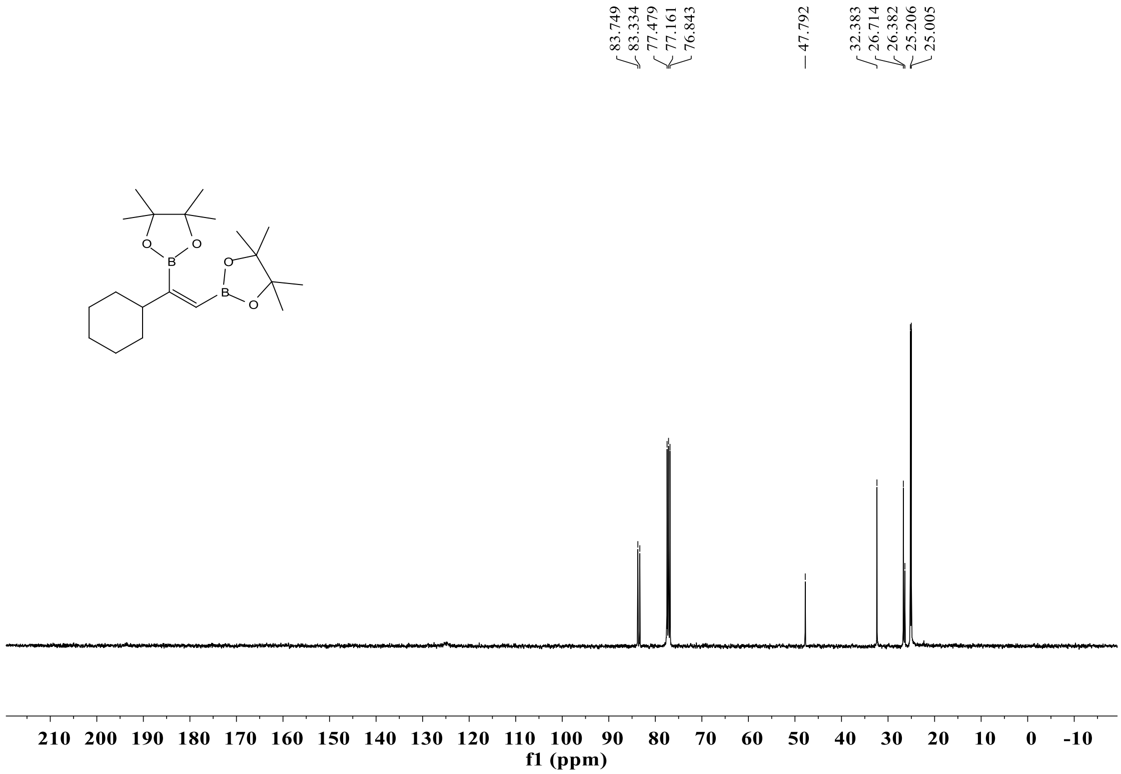

Supplementary Figure 35 | ^{13}C NMR (101 MHz, $CDCl_3$) spectrum of (*E*)-2,2'-(1-cyclohexylethene-1,2-diyl)bis(4,4,5,5-tetramethyl-1,3,2-dioxaborolane) (3ka)

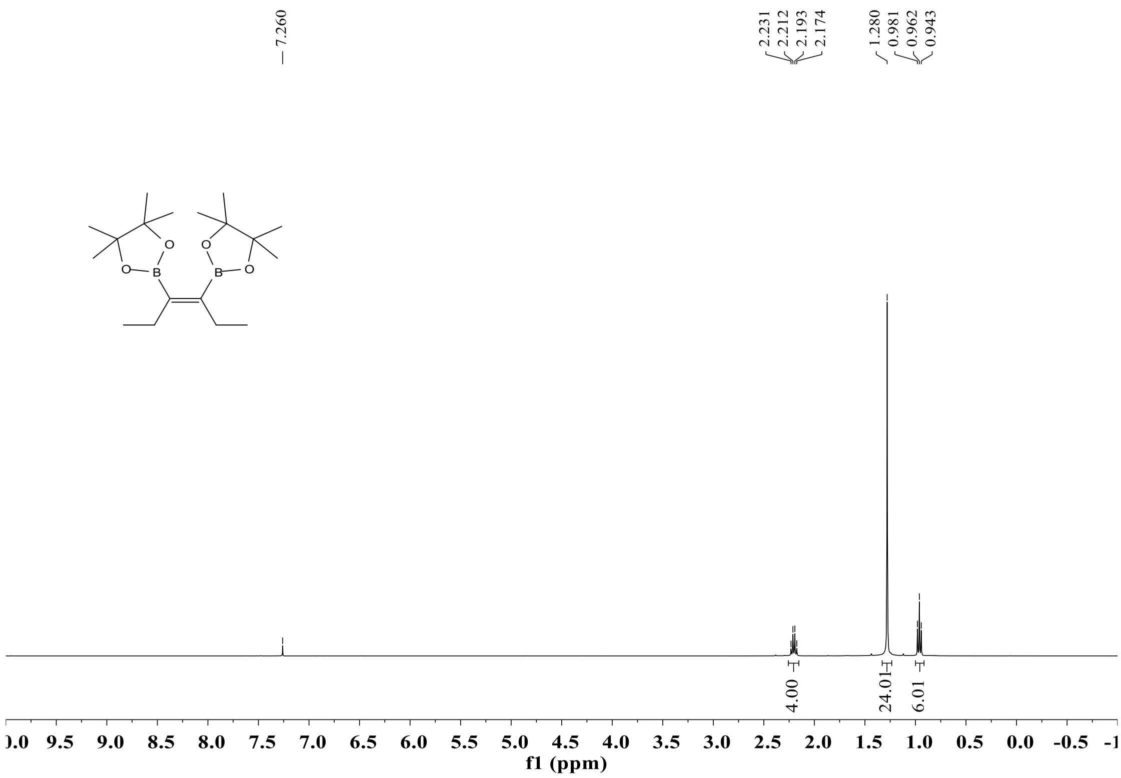

Supplementary Figure 36 | ^{1}H NMR (400 MHz, $CDCl_3$) spectrum of (*Z*)-2,2'-(hex-3-ene-3,4-diyl)bis(4,4,5,5-tetramethyl-1,3,2-dioxaborolane) (3la)

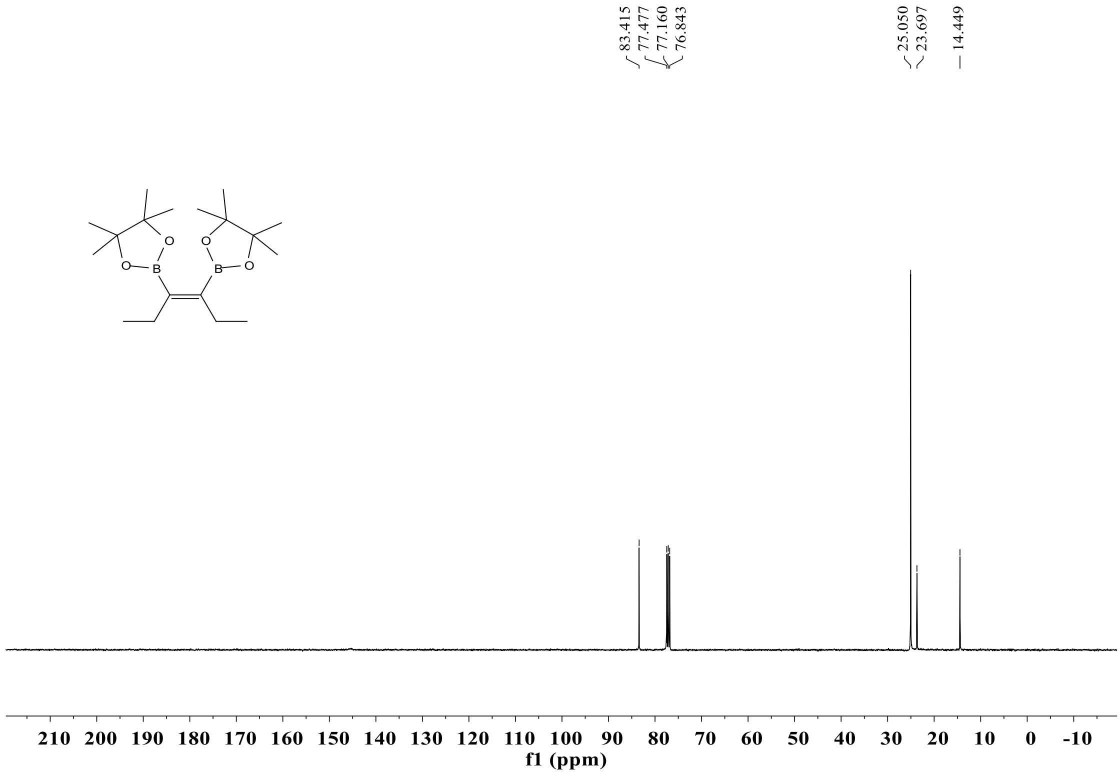

Supplementary Figure 37 | ^{13}C NMR (101 MHz, $CDCl_3$) spectrum of (*Z*)-2,2'-(hex-3-ene-3,4-diyl)bis(4,4,5,5-tetramethyl-1,3,2-dioxaborolane) (3la)

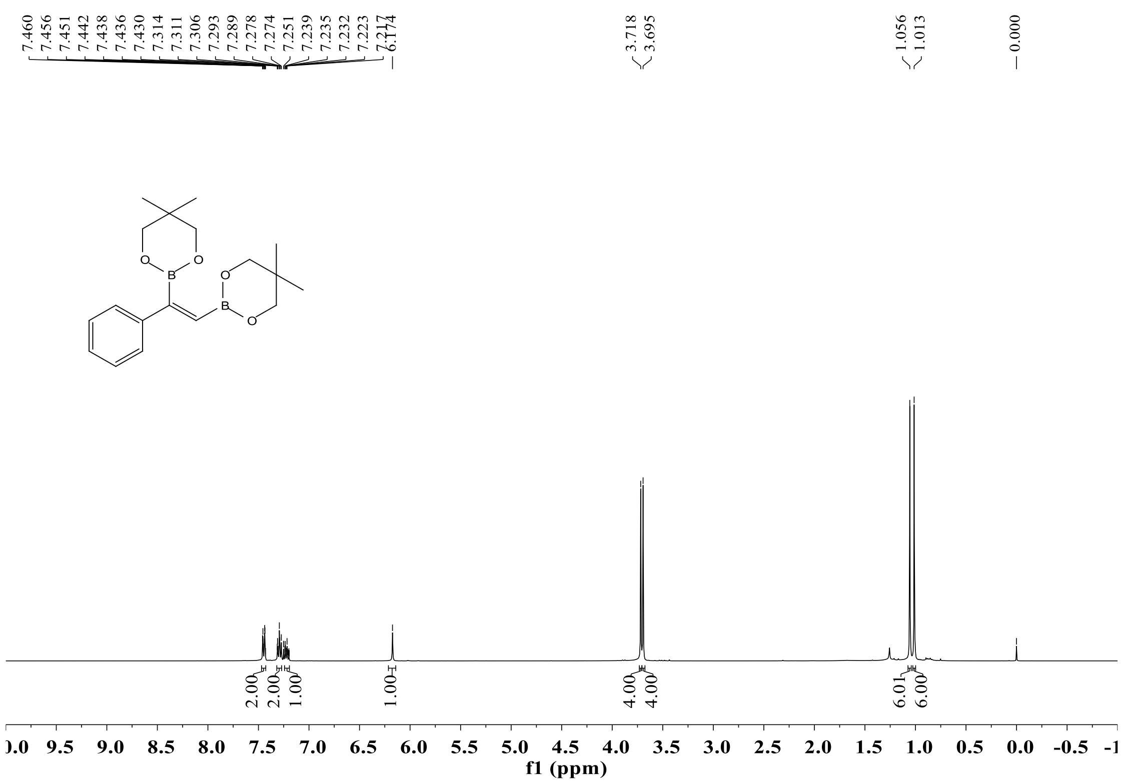

Supplementary Figure 38 | ^{1}H NMR (400 MHz, $CDCl_3$) spectrum of (*E*)-2,2'-(1-phenylethene-1,2-diyl)bis(5,5-dimethyl-1,3,2-dioxaborinane) (3ab)

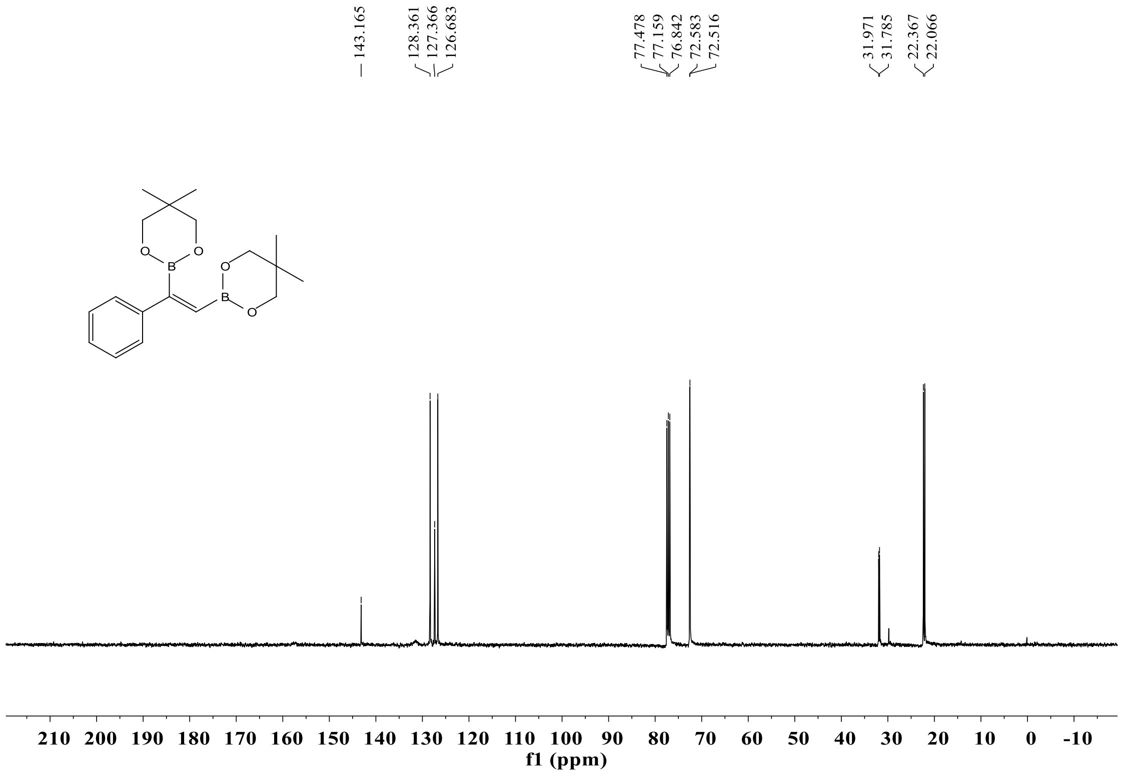

Supplementary Figure 39 | ^{13}C NMR (101 MHz, $CDCl_3$) spectrum of (*E*)-2,2'-(1-phenylethene-1,2-diyl)bis(5,5-dimethyl-1,3,2-dioxaborinane) (3ab)

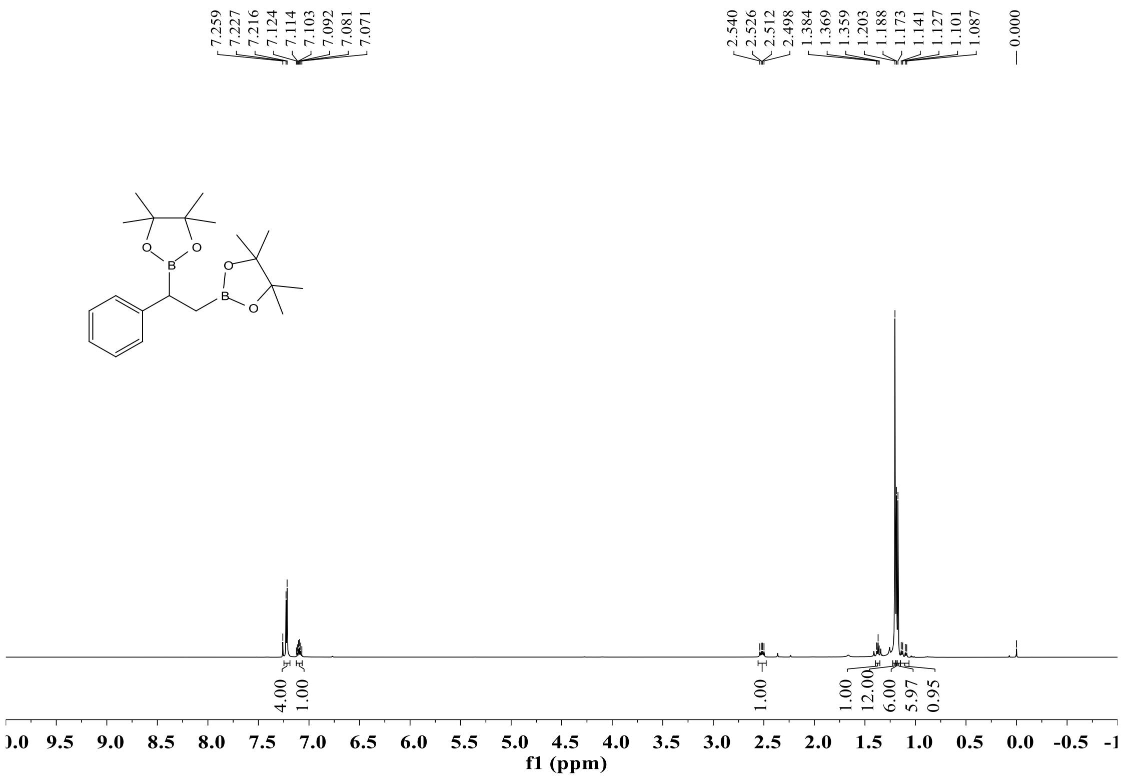

Supplementary Figure 40 | ^{1}H NMR (400 MHz, $CDCl_3$) spectrum of 2,2'-(1-phenylethane-1,2-diyl)bis(4,4,5,5-tetramethyl-1,3,2-dioxaborolane) (3ma)

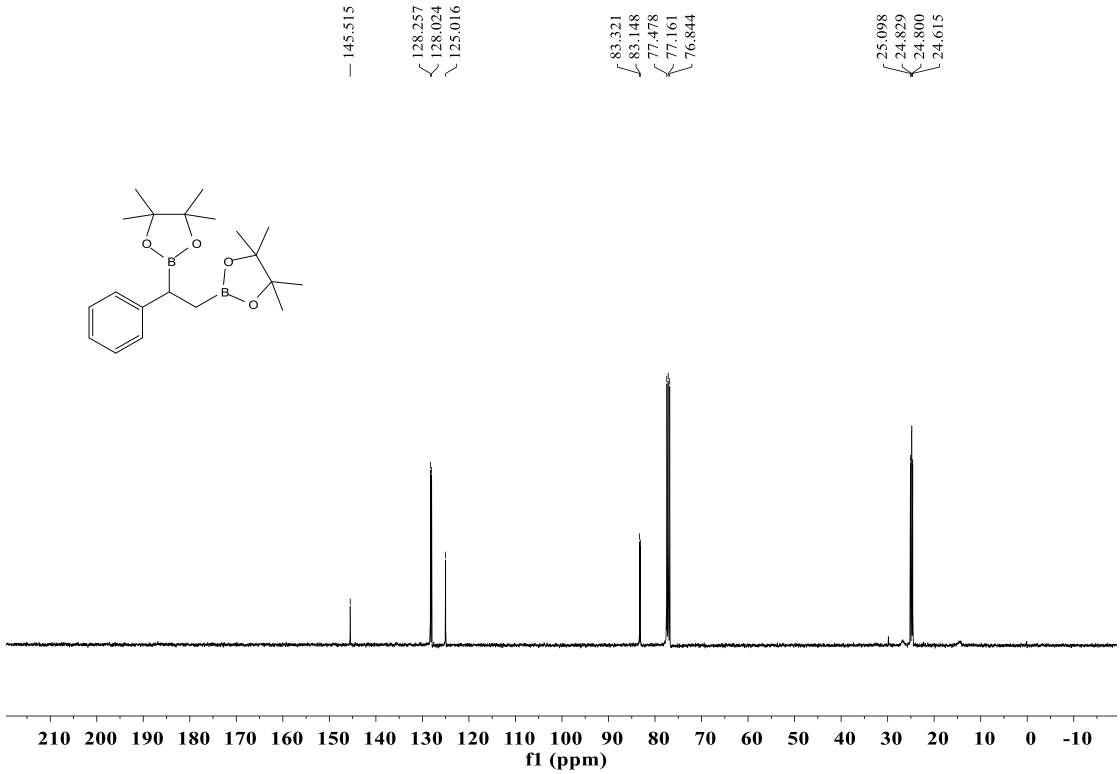

Supplementary Figure 41 | ^{13}C NMR (101 MHz, $CDCl_3$) spectrum of 2,2'-(1-phenylethane-1,2-diyl)bis(4,4,5,5-tetramethyl-1,3,2-dioxaborolane) (3ma)

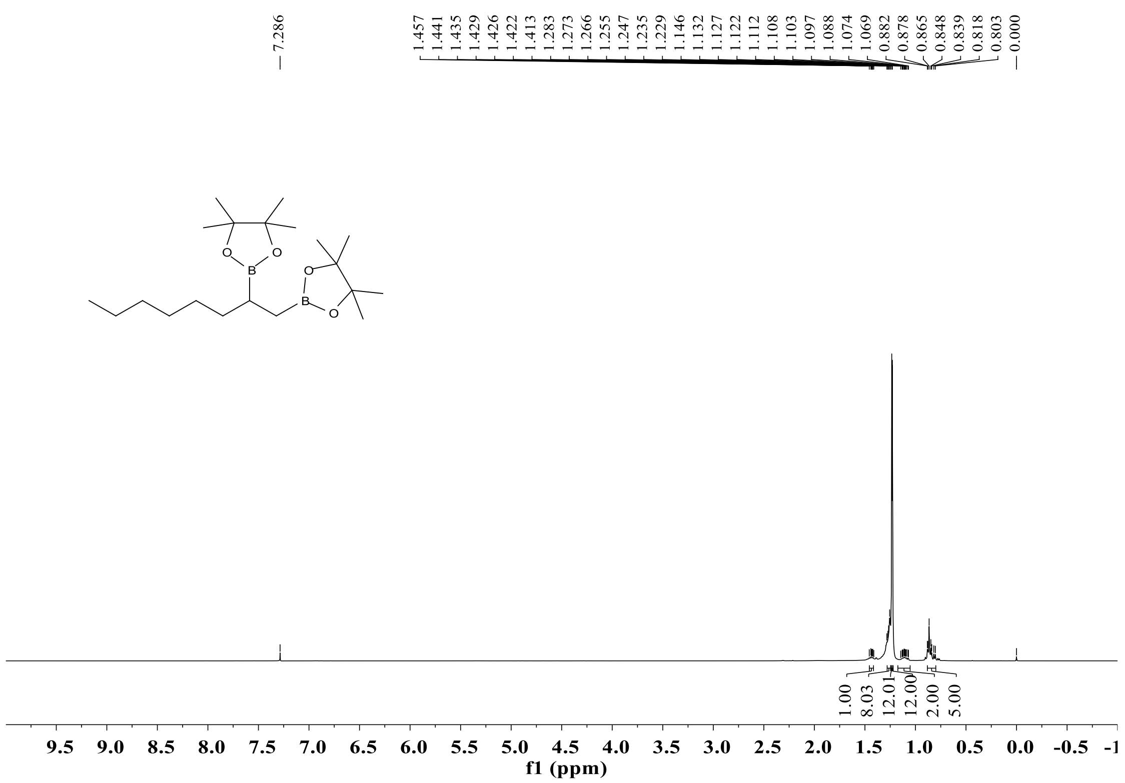

Supplementary Figure 42 | ^{1}H NMR (400 MHz, CDCl$_3$) spectrum of 2,2'-(octane-1,2-diyl)bis(4,4,5,5-tetramethyl-1,3,2-dioxaborolane) (3na)

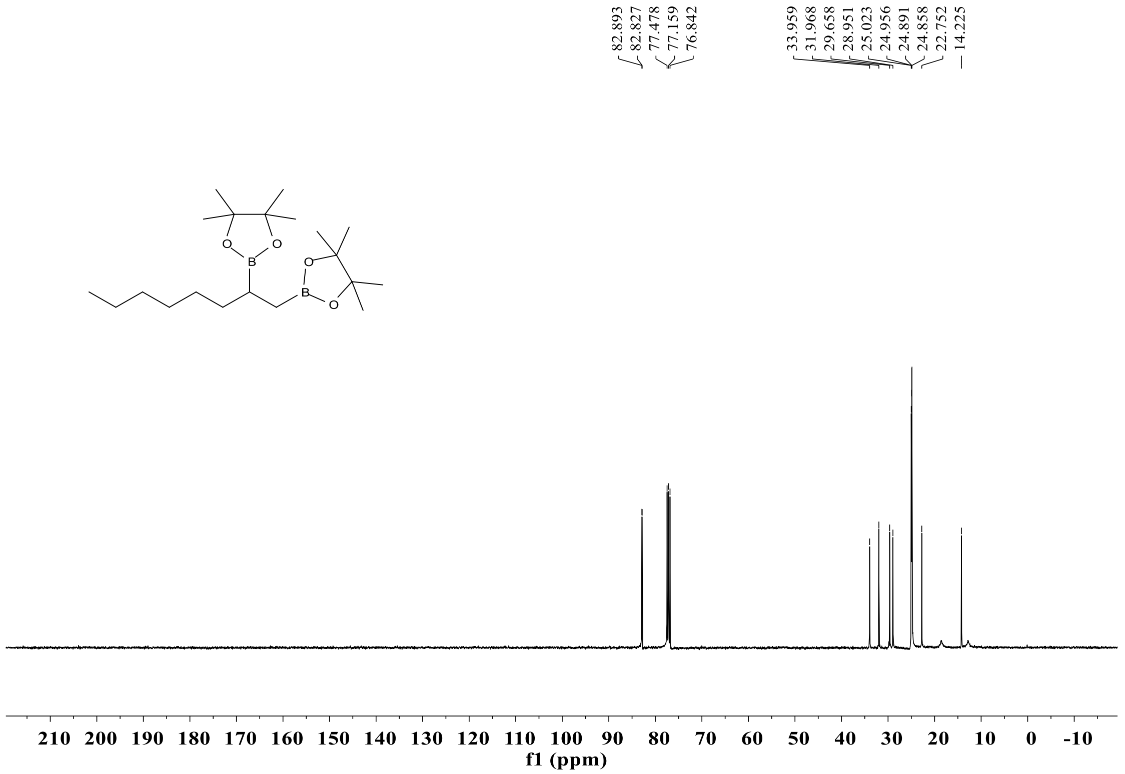

Supplementary Figure 43 | ^{13}C NMR (101 MHz, CDCl$_3$) spectrum of 2,2'-(octane-1,2-diyl)bis(4,4,5,5-tetramethyl-1,3,2-dioxaborolane) (3na)

Supplementary References

1. Fan, H. *et al.* Controllable Synthesis of Ultrathin Transition-Metal Hydroxide Nanosheets and their Extended Composite Nanostructures for Enhanced Catalytic Activity in the Heck Reaction. *Angew. Chem. Int. Ed.* **55**, 2167-2170 (2016).
2. Du, H., Jiao, L., Cao, K., Wang, Y. & Yuan, H. Polyol-mediated synthesis of mesoporous α-$Ni(OH)_2$ with enhanced supercapacitance. *ACS Appl. Mater. Interfaces* **5**, 6643-6648 (2013).
3. Yan, J. *et al.* Advanced asymmetric supercapacitors based on $Ni(OH)_2$/graphene and porous graphene electrodes with high energy density. *Adv. Funct. Mater.* **22**, 2632-2641 (2012).
4. Kresse, G. & Furthmuller, J. Efficiency of ab-initio total energy calculations for metals and semiconductors using a plane-wave basis set. *Comput. Mater. Sci.* **6**, 15-50 (1996).
5. Kresse, G. & Furthmuller, J. Efficient iterative schemes for ab initio total-energy calculations using a plane-wave basis set. *Phys. Rev. B* **54**, 11169-11186 (1996).
6. Blochl, P. E. Projector augmented-wave method. *Phys. Rev. B* **50**, 17953-17979 (1994).
7. Kresse, G. & Joubert, D. From ultrasoft pseudopotentials to the projector augmented-wave method. *Phys. Rev. B* **59**, 1758-1775 (1999).
8. Perdew, J. P., Burke, K. & Ernzerhof, M. Generalized gradient approximation made simple. *Phys. Rev. Lett.* **77**, 3865-3868 (1996).
9. Perdew, J. P., Emzerhof, M. & Burke, K. Rationale for mixing exact exchange with density functional approximations. *J. Chem. Phys.* **105**, 9982-9985 (1996).
10. Grimme, S. Semiempirical GGA-type density functional constructed with a long-range dispersion correction. *J. Comput. Chem.* **27**, 1787-1799 (2006).
11. Kamath, P. V. *et al.* Stabilized α-$Ni(OH)_2$ as electrode material for alkaline secondary cells. *J. Electrochem. Soc.* **141**, 2956-2959 (1994).
12. Freitas, M. B. J. G. Nickel hydroxide powder for NiO·OH/$Ni(OH)_2$ electrodes of the alkaline batteries. *J. Power Sources* **93**, 163-173 (2001).
13. Li, J., Zhao, W., Huang, F., Manivannan, A. & Wu, N. Single-crystalline $Ni(OH)_2$ and NiO nanoplatelet arrays as supercapacitor electrodes. *Nanoscale* **3**, 5103-5109 (2011).
14. Zeng, Z., Chang, K. C., Kubal, J., Markovic, N. M. & Greeley, J. Stabilization of ultrathin (hydroxy) oxide films on transition metal substrates for electrochemical energy conversion. *Nat. Energy* **2**, 17070 (2017).
15. Ishiyama, T. *et al.* Platinum(0)-Catalyzed Diboration of Alkynes with Tetrakis(alkoxo)diborons: An Efficient and Convenient Approach to cis-Bis(boryl)alkenes. *Organometallics* **15**, 713-720 (1996).
16. Takaya, J. & Iwasawa, N. Catalytic, Direct Synthesis of Bis(boronate) Compounds. *ACS Catal.* **2**, 1993-2006 (2012).
17. Ansell, M. B. *et al.* An experimental and theoretical study into the facile, homogeneous (N-heterocyclic carbene)2-Pd(0) catalyzed diboration of internal and terminal alkynes. *Catal. Sci. Technol.* **6**, 7461-7467 (2016).
18. Morgan, J. B. & Morken, J. P. Catalytic enantioselective hydrogenation of vinyl bis(boronates). *J. Am. Chem. Soc.* **126**, 15338-15339 (2004).
19. Alonso, F., Moglie, Y., Pastor-Perez, L. & Sepulveda-Escribano, A. Solvent- and Ligand-free Diboration of Alkynes and Alkenes Catalyzed by Platinum Nanoparticles on Titania. *ChemCatChem* **6**, 857-865 (2014).
20. Brown, S. D. & Armstrong, R. W. Synthesis of Tetrasubstituted Ethylenes on Solid Support via Resin Capture. *J. Am. Chem. Soc.* **118**, 6331-6332 (1996).

21. Khan, A., Asiri, A. M., Kosa, S. A., Garcia, H. & Grirrane, A. Catalytic stereoselective addition to alkynes. Borylation or silylation promoted by magnesia-supported iron oxide and cis-diboronation or silaboration by supported platinum nanoparticles. *J. Catal.* **329**, 401-412 (2015).
22. Bonet, A., Pubill-Ulldemolins, C., Bo, C., Gulyas, H. & Fernandez, E. Transition-metal-free diboration reaction by activation of diboron compounds with simple Lewis bases. *Angew. Chem. Int. Ed.* **50**, 7158-7161 (2011).
